# Perceptions of diabetes patients and their caregivers regarding access to medicine in a severely constrained health system: A qualitative study in Harare, Zimbabwe

**DOI:** 10.1371/journal.pgph.0000255

**Published:** 2022-03-03

**Authors:** Dudzai Mureyi, Nyaradzai Arster Katena, Tsitsi Monera-Penduka

**Affiliations:** 1 Faculty of Medicine and Health Sciences, Department of Biomedical Informatics and Biomedical Engineering, University of Zimbabwe, Harare, Zimbabwe; 2 Faculty of Medicine and Health Sciences, Department of Primary Healthcare Sciences, University of Zimbabwe, Harare, Zimbabwe; 3 Faculty of Medicine and Health Sciences, Department of Pharmacy and Pharmaceutical Sciences, University of Zimbabwe, Harare, Zimbabwe; BRAC University James P Grant School of Public Health, BANGLADESH

## Abstract

Nearly half of all sub-Saharan African countries lack operational Diabetes Mellitus policies. This represents an opportunity to build reliable evidence to underpin such policies when they are eventually developed. Representing the interests of those with the experience of living with the condition in national diabetes policies is important, particularly the interests regarding medicine access, a key pillar in diabetes management. One way to achieve this representation is to publish patient perceptions. Patient perspectives are especially valuable in the context of diabetes in Sub-Saharan Africa, where much of the empirical work has focused on clinical and epidemiological questions. We therefore captured the challenges and suggestions around medicine access articulated by a population of diabetes patients and their caregivers. This was a qualitative interpretivist study based on data from focus group discussions with adult diabetes patients and their caregivers. Eight FGDs of 4–13 participants each whose duration averaged 13.35 minutes were conducted. Participants were recruited from diabetes outpatient clinics at two health facilities in Harare. One site was Parirenyatwa Hospital, the largest public referral and teaching hospital in Zimbabwe. The other was a private for-profit facility. Ethics approval was granted by the Joint Research Ethics Committee for University of Zimbabwe College of Health Sciences and the Parirenyatwa Group of Hospitals (Ref: JREC 295/18). Diabetes patients and their caregivers are interested in affordable access to medicines of acceptable form and quality with minimum effort. Yet, they often find themselves privileging one dimension of access over another e.g. prioritising affordability over acceptability. Based on participants’ articulations, a sound diabetes policy should: 1. provide for financial and consumer protections, 2. regulate healthcare business practices and medicine prices, 3. provide for a responsive health workforce attentive to patient problems, 4. accord the same importance to diabetes that is accorded to communicable diseases, 5. decentralize diabetes management to lower levels of care, 6. limit wastage, corruption, bad macro-financial governance and a lack of transparency about how funding for health is used, and 7. provide support to strengthen patients’ and caregivers’ psychosocial networks. A diabetes policy acceptable to patients is one infused with principles of good governance, fairness, inclusiveness and humanity; characterised by: financial protection and price regulation, consumer protection, equity in the attention accorded to different diseases, decentralized service delivery, inclusion of patient voice in political decision-making, a responsive compassionate health workforce, psychosocial support for patients and their caregivers and allocative efficiency and transparency in public expenditure.

## Introduction

### Diabetes in Sub-Saharan Africa and the importance of patient perceptions

At least 340 million people are estimated to be living with Diabetes Mellitus (diabetes) globally and the cost of managing it exceeded half a trillion dollars as far back as 2015 [1]. In Sub-Saharan Africa (SSA) diabetes-related deaths occur mostly in people under the age of sixty [1], i.e. those in the economically active age group. In this region of the world, (where strained health systems are still grappling with high burdens of infectious disease), barriers to accessing diabetes diagnostic services, diabetes medicines and self-monitoring tools exist. Data and data collection systems on diabetes prevalence, morbidity and mortality are often unreliable, thus preventing proper estimates and hindering the crafting of appropriate responses and policies [2–4].

A national operational policy, strategy or action plan for diabetes is critical because it forms the basis for resource allocation and systematic intervention implementation in addressing this important global public health problem. Yet, by 2019, nearly half of all sub-Saharan African countries lacked operational diabetes policies or plans [5]. While this is a dire observation, it means there’s significant scope for SSA countries to improve. An opportunity exists to build a reliable evidence base that can underpin diabetes policies. These diabetes policies must be attentive to factors affecting access to medicines for diabetes patients because the key to effective diabetes management includes adherence to insulin and oral medicines [6,7]. Among the several frameworks conceptualising the access to medicines and health technologies [6–15], two of the most recently published [6,7] relate specifically to medicines for cardiovascular diseases and diabetes, suggesting the recognition of the unique salience of diabetes and cardiovascular diseases. ‘Access to medicine’ is defined here as the multi-dimensional construct that characterises the degree to which quality and safe medicines are available, affordable, accessible and acceptable in a timely manner to those needing them [6–15]. Beran et al., [6], described a framework for promoting diabetic patients’ access to insulin. These authors called for the synergistic efforts of governments, the pharmaceutical private sector and a civil society that includes a robust patient voice, as the necessary ingredients for ensuring access to insulin. Yet they noted that diabetes patients are rarely represented at top levels of diabetes policymaking. Amplifying patient voice bodes well for service delivery, patient experience and health outcomes [16] and one way to achieve this amplification is to conduct research, explore and publish patient perceptions. Patient voice though, is not always exercisable [17] and in Zimbabwe (one of the countries without a substantive diabetes plan [5]), citizens’ voice was found lacking at decision-making level [18].

The objective of this study was therefore to explore the lived experiences of diabetes patients regarding access to insulin and oral diabetes medicines in Zimbabwe. These perspectives, gathered from diabetes patients and their caregivers in Zimbabwe, (a low-resource setting in SSA), provide insights into what a national diabetes policy or plan in Zimbabwe might look like. Granted, insights from Zimbabwe cannot be assumed to be applicable to the rest of SSA. However, patient perspectives such as those presented in this paper, are valuable in the broader context of diabetes in SSA. They are valuable because they point to the idea that diabetes patients and their caregivers have articulable experiences that are relevant when crafting people-centred national diabetes policies in SSA, where much of the empirical work has focused on clinical and epidemiological questions [2] perhaps, at the expense of patient perspectives. While it is urgent to improve prevention, diagnosis, and treatment of diabetes in Zimbabwe and indeed SSA, is it equally important to seek patient perspectives. This is because patient perspectives provide clues to principles that patients feel should underpin an acceptable national diabetes plan.

### Diabetes and Zimbabwe’s health system

In Zimbabwe, a country with a population of 14.86 million [19], both the public and private sectors play roles in health financing and health service delivery [20]. Following independence from colonial rule in 1980, the Zimbabwean government focused on equitable access to health and made significant investments in public health infrastructure and interventions [21]. However, Zimbabwe’s health system started to deteriorate from the mid-1990s due to economic underperformance [22]. This impacted Zimbabwe’s progress towards Universal Health Coverage, a goal Zimbabwe has been aiming for since 2009 [23]. Coupled with the economic underperformance was a monetary policy that was associated with a weak local currency. This motivated the public and businesses to transact in United States (US) Dollars instead [24]. Pricing of medicines in US Dollars occurred. By 2015, household out-of-pocket (OOP) payments (24%) and external developmental assistance (25%) were the biggest health financing sources, whereas government spending comprised 21% of all health spending [25]. This inadequate government funding plus the mass emigration of skilled healthcare workers, have been cited as some of the causes of Zimbabwe’s critical health worker shortage. The density of physicians for instance, was recently reported to be 0.067 per 1000 population [22]. In terms of organization, the public health system comprises four levels of care. Each level of care is differentiated by the size of the facilities, the qualifications of the staff that works there, the scope of services that can be offered there and the types of medicines that can be stored or dispensed from there. The primary level consists of over 1 400 urban and rural clinics manned by nurses. The secondary and tertiary levels of care (that are designed to be accessed through a referral system that begins at the primary level), comprise forty-four district and eight provincial hospitals respectively. The fourth (quaternary) level of care comprises six referral hospitals [20]. Zimbabwe’s resource allocation system is skewed towards hospitals, with the higher-level provincial and central-level hospitals receiving more funds/subsidies than the lower-level clinics [26]. Zimbabwe has a diabetes prevalence rate estimated to be the highest among the most populous African countries. Projections indicate that Zimbabwe could have over 1.2 million diabetes patients by the year 2035 [27]. In addition, Zimbabwe exhibits the attributes of a fragile state [28–39] as described in fragility literature [40–42]. Fragility compounds health challenges because it undermines health service delivery and good health in general [40,42]. Research with diabetes patients in a context as constrained as Zimbabwe therefore satisfies the ‘the intensity criterion’, in qualitative research [43] i.e. using a case likely to produce a rich picture by virtue of the intensity of the problem or diversity of observations.

The immediate aftermath of the removal of Zimbabwe’s long-time head of government in 2017, was defined by optimism locally and internationally [44,45]. Certain corners of the global health community suggested that with the political change, a new national public health agenda was afoot [46] or ought to be [47,48], in order to resuscitate Zimbabwe’s health system marred by broader economic and health sector challenges that included disrupted medication supply [48–50]. The president of the Zimbabwe Diabetes Association was appointed Zimbabwe’s deputy health minister [51]. Afterwards, plans to implement the eponymous *Novartis Access* in Zimbabwe were announced [52]. *Novartis Access* provides selected low and middle-income countries with a basket of 15 medicines (including some diabetes ones) at the subsidised price of US$ 1 per treatment per month. An evaluation of *Novartis Access* after its first implementation year in Kenya showed no effect on medicine availability in households or on their price at health facilities [53]. This observation could have been partly due to a disconnect between the initiative’s implementation modalities and patient-related preferences [53]–preferences which ought to have been investigated and considered prior to implementation. It’s hoped that the articulations of diabetes patients presented in this paper will inform the *Novartis Access* implementation in Zimbabwe or other interventions intended to improve access to diabetes and noncommunicable disease therapies in similar contexts.

## Methods

This section describes our methods according to the requirements of the Consolidated Criteria for Reporting Qualitative Research (COREQ) [54] and the Standards for Reporting Qualitative Research (SRQR) [55].

### Ethics statement

Ethics approval was granted by the Joint Research Ethics Committee for University of Zimbabwe College of Health Sciences and the Parirenyatwa Group of Hospitals (Ref: JREC 295/18). Formal written Informed consent was sought and obtained from participants before data collection commenced.

### Research philosophy

This study was grounded in the critical realism philosophy. Critical realism is the philosophy of science that conceives reality as stratified into three realms: the ‘empirical’, the ‘actual’ and the ‘deep’ [56] (ontological position). The ‘empirical’ contains observable phenomena in the positivist sense e.g. experiences and actions (e.g. diabetes patients in an outpatient clinic waiting area exhibiting signs of diabetes complications). The ‘actual’ consists of events that happen or exist but are not readily empirically accessible, (e.g. poor access to diabetes medicines). Researchers might however observe these events through focused data collection and analysis (e.g. conducting FGDs with patients) [57]. The ‘deep’ comprises things or causal pathways/mechanisms that produce phenomena in the empirical and actual realms. These things in the ‘deep’ cause observable reality either by being present or even by being absent (e.g. the absence of a sound national diabetes policy or the presence of difficult macroeconomic conditions inhabit the deep realm and cause observable poor access to diabetes medicines). Different causal pathways may be activated or inactivated by contextual factors [58–60]. Context therefore matters in critical realist research, because it determines which causal pathways are activated and which are not. Case study research, an intensive research design suited to uncovering causal explanations in contexts, [61] is therefore particularly compatible with critical realist research [62]. This was this paper’s study design.

Critical realism embraces interpretivist paradigms because it holds that the knowledge and the interpretation of reality is always influenced by the subjectivity of people (both researchers and participants) [63]. Because knowledge construction has these subjective aspects, and the activation of causal powers that produce effects is dependent on context, critical realists accept that knowledge of reality is essentially provisional and fallible. It’s subject to incremental modification as more discoveries occur [58] (epistemological position). Therefore it is accepted that the findings documented in this paper may not be applicable to other SSA health systems where different researchers and diabetes patients occupy and where different causal pathways are activated. These findings may not even be applicable to Zimbabwe in future, during a different time period when different contextual factors are activated.

Finally, human emancipation and the improvement of society are guiding values for critical realists [59,64] (axiological position). Critical realism’s focus on uncovering mechanisms of how things do happen, combined with its (poststructuralist) stance that views humans as capable of transforming society [56,65] justifies making policy recommendations for how things ought to happen in order to improve society (as this paper does by recommending crafting patient-sensitive diabetes policies) [58].

### Study design

This study was an interpretivist qualitative case study. A case is a phenomenon occurring in a spatially, temporally or demographically bounded context and is a unit of analysis in research [43]. A case study is therefore described here as the study of a phenomenon in a single (Zimbabwean) context as it unfolds/unfolded naturally (as opposed to study by experimental design). It employed the analysis of text derived from focus group discussions (FGDs) with diabetes patients and their caregivers (family members). The interpretivist paradigm, as well as qualitative research methods, embody unique qualities that made them suitable to researching the complex and varied subjective perceptions of diabetes patients in the study context. With interpretivist qualitative research, people’s lived experiences and the subjective interpretations they attach to those realities, are valid contributions to research and knowledge. No one individual’s reality is privileged over another’s. With the qualitative interpretivist approach, the goal is to provide rich (often diverse) contextualised insights as opposed to generalised laws that are upheld in every context. Interpretivism therefore utilises, as we did, in-depth formal group discussions and/or interviews to elicit rich and thick descriptions of reality from the perspectives of participants. Furthermore, analysis of findings in interpretivist research acknowledges that researchers’ subjectivity also influence their interpretations of the data. This allows researchers to be reflexive, to consciously interrogate their own biases and to seek ways of validating their conclusions [66]. One of the ways this paper’s authors validated their interpretations was to ensure that all three researchers independently analysed and coded all the transcripts, before meeting to discuss their coding and interpretations. The conclusions are based on the interpretations that all the three authors considered amply supported by the data.

### Research team and reflexivity

At the time of the study, the research team comprised three female researchers trained in qualitative research at postgraduate level. DM and TMP are registered pharmacists and NAK is a public health practitioner. TMP holds a Ph.D. in Clinical Pharmacology and at the time of data collection and analysis, DM and NAK were undertaking doctoral research looking at pharmaceutical systems and diabetes care. DM received training in Research Data Management and Data Protection. The participants had no prior acquaintance with any of the researchers and none of the researchers were involved in the care of patients at the sites where data was collected. Apart from academic interest, the researchers had no other interest in the study. There was no further contact with participants after the conclusion of each FGD. Before conducting the FGDs, based on the researchers’ familiarity with the Zimbabwean context, the researchers anticipated that participants would articulate issues to do with the poor availability, affordability and accessibility of oral medicines and insulin. This expectation was met. However, what wasn’t expected was how impassioned and vivid these articulations would be. This observation made the researchers more sensitive to themes such as empathy, justice and fairness, during data collection and analysis. It also hadn’t been expected that paradoxical testimonies about access challenges would be uncovered. For instance, as described in the results and discussion sections of this paper, authors were surprised to discover that patients with health insurance were actually more financially burdened than uninsured ones. Prior to data collection, the researcher (DM), a pharmacist by profession, had considerable preconceived ideas and prior knowledge about the research subject and what caused diabetes patients’ constrained access to medicines. In order to limit the undue influence of this prior professional knowledge on the data collection, DM sent weekly group emails to her two doctoral supervisors who were neither Zimbabwean residents nor health professionals. They were experienced qualitative researchers but were more distanced from the research phenomenon. In these long reflexive emails, DM documented key questions and observations in the FGDs. In response, the supervisors provided feedback that facilitated the identification of blind spots and biases (and good practices). These academics also reviewed the FGD guide before use and identified leading questions that suggested DM’s bias. As Mauthner and Doucet pointed out, such regular interactions with one’s research group/team significantly enhance a researcher’s ability to be reflexive [67].

### Sampling location and criteria

This study was conducted in Harare, Zimbabwe’s capital, for reasons related to logistical convenience. In addition, compared to other locations in Zimbabwe, Harare is the location of institutions/actors involved in Zimbabwe’s pharmaceutical system (pharmaceutical wholesalers and manufacturers, the only two universities that train pharmacists, high-level Ministry of Health officers, office bearers of pharmaceutical professional associations etc.). Therefore, Harare represented the most complete microcosm of Zimbabwe’s pharmaceutical system. Participants were recruited from diabetes outpatient clinics at two health facilities in Harare. These were:

- Parirenyatwa Hospital, the largest public referral and teaching hospital in Zimbabwe and
- Centre for Diabetes Management, a private for-profit facility.

These study locations were selected for their accessibility as well as the ease of recruiting participants there. Having one study site that is a public hospital and another that is a private for-profit facility increased the possibility that a heterogenous sample (in terms of socioeconomic status), would be recruited. This was considered advantageous because diverse testimonies were being sought. These sites held diabetes outpatient clinics where diabetes patients and/or their caregivers presented, congregated and participated in support group discussions. Based on residential address data, patients who visit Parirenyatwa Referral Hospital reside in diverse geographical regions of Zimbabwe [68]. The same is true for the private for-profit facility. It was noted that several peer-reviewed studies focusing on diabetic populations in Zimbabwe recruited solely from Parirenyatwa Hospital [68–72]. Participants were eligible for inclusion in the study if they were adults on diabetes medication or caregivers/guardians of such.

### Data collection and management

Data was collected by one researcher (DM) through audio-recorded (FGDs) with patients recruited at the outpatient clinics’ waiting area. Each FGD was conducted at a secluded location away from the waiting area to protect participant privacy, protect the quality of the audio recording and pose minimum disruption to the clinic workflow. FGDs helped to triangulate and validate the testimony of participants in real-time as participants compared experiences. Sampling was purposive, based on the inclusion criteria already stated and willingness to participate. Potential participants were publicly addressed in the outpatients’ waiting areas and willing FGD participants interviewed separately. This recruitment procedure made it impossible to determine the number of eligible participants who declined to participate. After consenting, no participant withdrew from the FGD although it was made clear during the consent-seeking process that they could withdraw if they so wished. Because FGDs were conducted with patients who happened to be in an outpatient clinic on the days of data collection, who resided in different parts of the country, member-checking was impractical. However, during the FGDs, the researcher occasionally asked clarifying questions when ambiguous or surprising testimony was given. The guides used by the researcher to moderate the FGDs (attached as S1 and S2 Files) were tested in the first FGD group and no changes were subsequently made. Eight FGDs of 4–13 participants each whose duration averaged 13.35 minutes (excluding the time the moderator spent on opening and closing remarks), were conducted between 27 February 2019 and 20 March 2019. No follow-up FGDs with the same participants were conducted.

FGDs were conducted until what is commonly understood as ‘saturation’ was achieved. Saturation is a problematic concept as multiple researchers have varying ways of defining it, measuring it or determining when it’s been achieved [73]. Furthermore, it is impossible to fully rule out the discovery of new insights with continued data collection [74]. This dilemma was resolved by ceasing data collection when it was considered that further data collection would offer increasingly diminishing returns given the consistency of testimony from the FGDs already conducted (ibid). Practical and ethical considerations also contributed to the cessing of data collection when the testimony became consistent with each subsequent FGD. Such practical and ethical considerations included: the finite resources available for data collection and analysis, and the reluctance to keep disrupting the weekly outpatient clinic activities simply to recruit participants. All transcripts of audio-recordings were checked for completeness and accuracy by all the researchers. Some of the text in the transcripts was in the vernacular language of ChiShona and was analysed in this form by all the researchers, who have expert level proficiency in both English and ChiShona. Only ChiShona excerpts included in this paper were translated into English. Translations were discussed by researchers to determine, by consensus, that the original essence of each translated excerpt had been retained. No personal identifying information was contained in the audio recordings or transcripts. All project files were stored on the researchers encrypted and password-protected laptops. Transcripts of the FGDs are attached to this paper as S1 Data.

### Ethical considerations

Before being invited to sign the informed consent forms, participants had the study explained to them in ChiShona, within a group context. Then, individual participant information sheets were distributed to each participant. Those who had questions asked the researcher (DM) individually. Separate consent both for participation in the research and for audio recording of testimony was obtained from each participant. Participants were offered $5.00, which was sufficient for a modest lunch meal (according to the Zimbabwe National Statistics Office’s economic data at the time). This was offered as a token to appreciate time lost while participating in the research–time they could have been engaging in income-generating activities. While the ethical implication (coercion), and the methodological implication (selection bias) of incentivising participants are acknowledged, this token is generally a requirement in the research context. Local ethics approval would not have been granted in the absence of this provision in the study protocol. The token was kept small to minimize its persuasive power. Participants were given an option to decline this token and had to indicate on the consent form whether they had accepted or declined it. None declined.

### Analysis

FGD Transcripts were managed using NVivo 11 and MS Word computer programs. Analysis was deductive, using a pre-existing analytical framework described in the paragraph immediately following this one [9]. Each author listened to all recordings and deductively coded all transcripts solo, using the coding sheet derived from the analytical framework (S1 Table). DM then compared all three sets of coded transcripts side by side, noting differences in coding, which were subsequently discussed and resolved by consensus. Text that was unable to be categorised in any of the deductive codes was coded as ‘other’ and discussed to determine by consensus if new inductive codes were warranted. No new codes were warranted thus themes were identified deductively. How these themes resonated with extant literature on diabetes patient perspectives, is discussed in the discussion section of this paper.

#### Analytical framework

**Fig 1:**
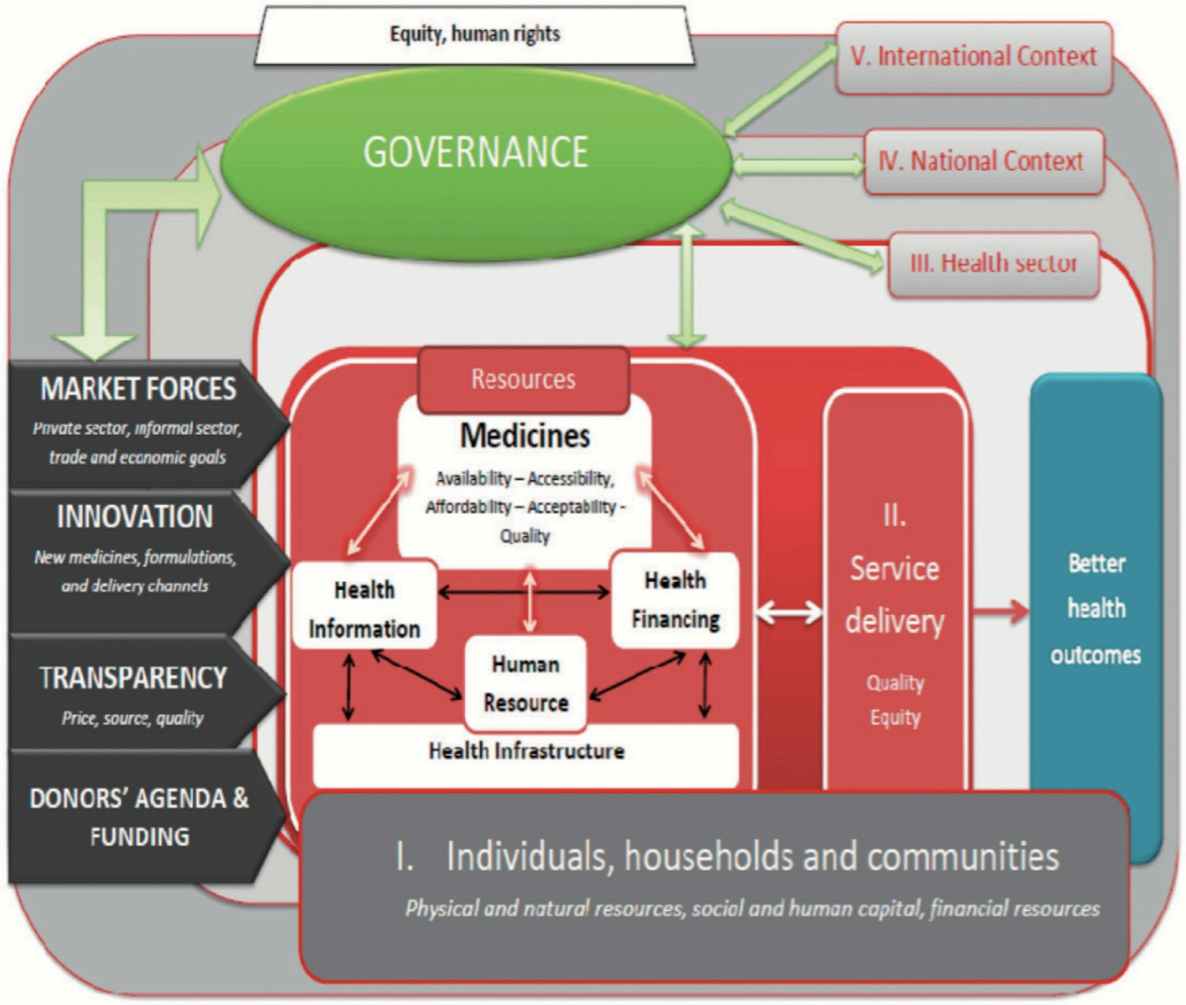
Access to medicines from a health system perspective [9]. This image was made available under the Creative Commons CC-BY-NC license and permitted non-commercial use, distribution and reproduction in any medium, provided the original work is properly cited.

Bigdeli et al. [9], advanced a nuanced way of conceiving ‘Access to medicine’ from a health system perspective. They considered multiple levels of organization from the household level to the international context (Fig 1). Among a set of extant access to medicines frameworks [6–15] this framework (Bigdeli et al.’s framework [9]) was found to be the most comprehensive conception yet of what access to medicine entails. Therefore, this framework’s domains formed the basis of our coding scheme outlined in S1 Data. The four domains in that framework are as follows: 1. **Individual Household and Community factors**; 2. **the Dimensions of Access** (i.e. availability, affordability, accessibility, acceptability and quality); 3. **Health System Building Blocks** [75] (i.e. Financing, human resources, health information, service delivery and supply chain), and 4. **Cross-cutting factors** (i.e. Equity, Governance, Transparency, Innovation, Donors’ Agenda and Market forces).

## Results

All FGD participants were literate male and female adult patients or adult caregivers/guardians of diabetes patients. Focus group discussions were conducted at both a public referral hospital and at a private diabetes clinic.

**Public hospital Respondents Profile:** During data collection, with the exception of outpatients under the age of 5 or over the age of 65 who were treated for free, outpatients at Parirenyatwa hospital were charged US$15.00 in user fees [76]. This was significantly cheaper than the private sector and as a result, low-income households tended to seek care there [77]. Based on FGD testimony, some of which is contained as excerpts in the results section of this paper, participants recruited at Parirenyatwa hospital came from low-income households, were generally uninsured, used public transportation to travel to the hospital and walked on foot in search of medicines and bargain prices at various pharmacies.

**Private clinic Respondents Profile:** By comparison, participants recruited at the Centre for Diabetes Management were more economically well-off. The user fees there were considerably higher, (US$120 charged to first-time patients and US$70 charged for each subsequent visit). Based on FGD testimony, participants recruited at this site were generally insured. They owned and drove (or were driven in) personal vehicles when searching for medicines at pharmacies. They also understood the value of being represented at diabetes policymaking platforms.

The views expressed by the participants, such as the one illustrated below, indicate that individuals suffering from diabetes have considerable appreciation for the seriousness of diabetes as a life-threatening condition:

*“Because of our diabetes, we are close to death. We can die anytime. The life of a person with AIDS is actually different from ours”. **Text F in S1 Data** [Quote IHC1]*

The role of adherence to correct regimens of medicines from trusted sources, blood glucose monitoring and dietary modifications, in effectively managing diabetes was understood. See one exemplary quotes below and the rest [IHC1-IHC6] in S2 Table.

*“I wasn’t taking diabetes medication for 5 days* ***therefore*** *my blood sugar levels rose”*. ***Text B in S1 Data*** [*Quote* IHC2]

The expectation of representation at policy-making platforms (since the deputy health minister is the former president of the Zimbabwe Diabetes Association) was also well articulated by diabetes patients recruited from the private sector facility.

*“But why are we falling short? Our patron is the deputy [health] minister surely… our patron being a diabetologist and a deputy [health] minister…I was happy when he was appointed deputy [health] minister, thinking now our problem is resolved… But we seem to be getting worse.” **Text H in S1 Data** [IHC7]*

We suggest that taken together, these quotes indicated that diabetes patients in the study context were generally well-informed about the outpatient management of their condition. They also saw a relationship between having their interests represented at ministerial (policy) level and having their grievances addressed by government.

This sections below contain the results of our deductive analysis. Exemplary supporting quotes from the data are contained in the narrative. Results are arranged thematically in four major sections associated with the four domains in the framework depicted in Fig 1 [9]. First, individual household and community factors are described, followed by the dimensions of medicine access (Availability, Affordability, Accessibility, Acceptability, and Quality), then Health system building blocks (Financing, Human Resources, Services, Information, Supply Chain) and finally the cross-cutting factors (Donors Agenda, innovation, transparency, market forces, Governance and equity).

### Individual and household factors

Discussions with participants who take care of diabetes patients revealed that the main individual and household factor affecting access to diabetes medicines and other treatment modalities is income (or lack thereof). Furthermore, caring for people on diabetes treatment often strains household income and stretches psychosocial support networks. This can then strain relations and mental health, as the quotes below illustrate.

*“You just sacrifice in order to buy the medicine but you won’t be having the money. Like myself, I don’t work, but I’m my mother’s caregiver. I am someone else’s dependent. So I then ask that someone else, for money to buy medicines for my mother”. **Text A in S1 Data***

*“This particular medicine is very expensive (holding up a packet of Glicliazide)….So here [at the public hospital], you won’t find it. You might find Enalapril, Nifedipine, HCT and metformin but the expensive ones, it’s hard. Such that at the end of the month you become stressed truly, I end up developing high blood pressure too”. **Text D in S1 Data***

*“One of the challenges is that, if you tell a diabetic family member that there’s no money [for medicines or food] they don’t understand.”*

Another participant in the same FGD session then agreed with this sentiment:

*“If you then go on to buy something else, they ask, ‘So there’s no money, huh?’” **Text C in S1 Data***

These quotes emphasise how diabetes (and possibly many other illnesses) is not merely a clinical condition that affects a patient, but can be a social issue that undermines household harmony and caregivers’ mental health.

### Dimensions of access: Availability, affordability, accessibility, acceptability and quality

#### Availability

Regarding the availability of medicines, all discussants recruited from the public referral hospital described the erratic availability of diabetes medicines there, particularly the expensive ones.

*“Here [at the public hospital], you may get the cheap ones. The expensive ones they say they don’t have. It’s a problem.” **FGD2***

The problems associated with the unavailability of medicines at the public hospital were compounded by the unavailability of particular oral medicines at private pharmacies. This unavailability of certain medicines in the private sector (particularly the fixed dose combination medicines), was also reported by participants recruited from the private diabetes clinic:

*“Medicines are a problem. For me, I take Galvus 50/1000. It’s unavailable. I haven’t seen a [private] pharmacy that has it. The pharmacist then suggested that I take the 50/500 and then take metformin 500 separately. That’s how I have done it for this month because 50/1000 is not available wherever you go.” **FGD8***

#### Affordability

When medicines and blood glucose testing strips were available in the private sector, their unaffordability was almost universally acknowledged by participants. Some even asked the FGD moderator if she knew of places they could access cheaper medicines:

*“We are taking our medicines but it’s expensive. Don’t you know somewhere where they are cheaper? We have been taking diabetes medicines for years but they have now become unaffordable.” **FGD6***

What made the medicines unaffordable for many was the fact that some medicines and insulin formulations were being sold by pharmacies in foreign currency (United States Dollars), which participants could not easily acquire:

*“I inject insulin and it’s hard when you don’t have US Dollars. To buy it, you’re told to bring US Dollars.” **FGD8***

It was not just the medicines themselves that made diabetes treatments unaffordable. Several of the participants resided outside Harare, the capital city, yet they had to collect their medicines at the public referral hospital in Harare (where this study was conducted). The transaction costs associated with traveling to the referral hospital to refill prescriptions made the whole endeavour unaffordable for them.

*“We come from afar. We don’t live here in Harare. We pay transport fares”. **FGD6***

This unaffordability of medicine access fuelled the tendency for patients privilege affordability over the acceptability of the service provider:

*“You, buy where it’s cheaper regardless of how you’re treated there”. **FGD5***

#### Accessibility

In order to find pharmacies where medicines were both available and affordable, most of the participants reported that they expended effort searching for medicines at multiple pharmacies and comparing prices. This process, in our view, compromises the accessibility of pharmacy services.

*“Last week I spent the whole day walking, I don’t think there’s a pharmacy I didn’t check until I went to [name of private pharmacy] where I was referred to [name of a different private sector pharmacy]”. **FGD6***

#### Acceptability

Regarding the acceptability of diabetes medicines available in their context, there were isolated responses that indicated some participants’ preference for oral medicines over injectable insulin. Likewise, the relative unacceptability of insulin administered by syringe and needle compared to pen-sets was articulated.

*“My father injects insulin. Is there a possibility that he can stop the injections and take oral tablets instead?” **FGD6***

*“The Pen-set is good because it’s easy to adjust the dose in millimetres. I just tune until it gets to the correct dose then inject myself. But with the syringe it’s harder. Sometimes at night the lighting is poor. You then just estimate.” **FGD2***

#### Quality

Concerning the quality of medicines, one participant indicated that he cared less about the quality of the medicines he procured as long as they accessed the medicines. His experience described in the quote below for instance, was recounted when the FGD moderator asked the participants about their confidence in the quality of the medicines they accessed:

*“Since I had walked around searching for long, I wanted to quickly get home. I was tired and I just trusted the pharmacist that what he had dispensed for me was ok.” **FGD2**.*

The majority of the participants however, expressed considerable trust in the quality of medicines dispensed at licensed pharmacies:

*“Since I’ll be looking only in pharmacies, I just trust that the pharmacy provides good quality medicines.” **FGD1***

This trust was expressed despite significant dissatisfaction with pharmacists’ business practices or in isolated cases, their clinical judgement (see results section on market forces further down in this paper’s results section). On one hand this is a positive observation; patients ought to have full confidence in the quality of medicines dispensed from health facilities. On the hand however, this unquestioning trust could be harmful if falsified, counterfeit medicines and substandard or expired medicines are

### Health system building blocks: Financing, human resources, service delivery, information, supply chain

#### Financing

Based on the testimony of the majority of the FGD participants, exemplified by quotes below, diabetes patients recognise the need for sustainable financing of medicine access through health insurance or government subsidies:

*“But health insurance subscriptions are less than what you will then fork out of pocket…*.*so I suggest you join a health insurance scheme…”* **FGD1 (participant advising another participant in the same FGD session)**

*“I think the government should do as it does for HIV medicines and supply medicines for free to diabetic patients*.*”* ***F*GD5**

We discovered that government spending on diabetes medicines is not evident in the wake of stock-outs at public institutions where user fees were charged. We also discovered that health insurance occasionally fails to deliver on the promise of financial protection for a variety of reasons. Some insurance plans are rejected outright by service providers. Some plans require co-payment in cash from the patient at the point of care. Some plans require that patients pay for medicines out of pocket, in forex, then claim for reimbursement in local currency, (calculated at exchange rates that are well below market ones). Furthermore, some service providers appear to fraudulently submit insurance claims to the insurers for the full cost of medicines after having collected the full amount in cash as co-payment from patients. The three quotes below, from the submissions of insured participants, illustrate all this.

*“Another thing I noticed with health insurance*, *ever since the confusion with currencies began*, *pharmacy personnel lie and claim that they accept health insurance*. *They make you fill the form and charge you a cash co-payment that’s the same as the full cash price in a different pharmacy*. *So I don’t know if we’re being defrauded or not”*. ***F*GD8**

*“Now*, *health insurance*, *sorry let me talk*, *pharmacies are not accepting health insurance*. *They ask you to pay out-of-pocket*, *then [advise you to go and] claim for reimbursement [from your insurer]…*. *You might buy your medicines in US Dollars but the health insurer doesn’t use the going exchange rate [when they reimburse you]*. *They treat the local currency and the U*.*S dollar as equivalent so they reimburse you in the local currency*. *That’s what is happening with medical aid*. *Useless*!*”* ***F*GD8**

*“Insulin in local currency so far is available but the issue is that we pay co-payments at the point of sale*.*”* ***F*GD1**

#### Human resources

On the human resources front, the desirability of ethical, compassionate, patient and helpful conduct by pharmaceutical service providers was frequently expressed in FGDs:

*“People of our mothers’ ages need to properly understand*. *Sometimes*, *they don’t even know the names of their medicines*. *So the health worker who attends to them needs to be patient and explain to them how things are*.*”* **FGD5**

*“Some medicines are locally manufactured and pharmacies purchase them in the local currency. But they end up retailing those medicines in US Dollars. So I think some of this behaviour is caused by cruelty. We no longer value patients’ lives. I don’t know, maybe I’m wrong.” **FGD2***

*“Pharmacy personnel is being duplicitous*. *The don’t dispense medicines for the full month…*.*I remember in February I bought medicines for a full month and they gave me enough for half a month”* ***F*GD2**

#### Service delivery

Participants showed they already appreciate the value of modern medicine in the management of diabetes but are dissatisfied with the inconvenience of having to travel to the central hospital for service delivery. Also dissatisfying about service delivery was the rationing of supplies that occurs at public hospitals, which prevented patients from purchasing more than one month’s supply of medication at a time:

*“Sometimes*, *when I find money*, *wanting to buy two bottles of my medicine*, *they tell me they can only dispense one [because of rationing]*, *but I would have paid 20 dollars for a round trip bus fare…”* ***F*GD5**

Asked why travelling to the referral hospital was necessary, one participant revealed that it was because local health facilities nearest to patients’ homes were more expensive than the tertiary hospital:

*“At my nearest hospital*, *just for consultation they charge 20 dollars*. *Here I pay 10 instead*.*”* ***F*GD5**

#### Health information

Access to information is an essential aspect of access to medicine. Information to do with where particular medicines can be sourced, therapeutic information and information about home management of diabetes appeared to be appreciated by nearly all participants:

*“What should we do when we can’t find medicines here*? *You [health professionals] must inform us where to go when we can’t find medicines here*.*”* ***F*GD6**

*“The doctor we met at today’s clinic properly explained things to us*. *After he’d written the prescription he explained to us what each medicine is for*. *So*, *that’s information that we appreciate*.*”* ***F*GD3**

Surprisingly, however, information about possible side effects related to medicine regimens was unwelcome because it was viewed by many respondents as a potential deterrent to adherence:

*“I only want to know that I’m being treated*. *If I’m told about the unpleasant effects of the medicine would I still want to take it*? *I don’t want to know*.*”* ***F*GD3**

We noted that State regulatory institutions, (in particular the consumer protection and national medicines regulatory agencies), as well as diabetes patient associations can do more to provide information regarding their mandates to the lay public. We arrived at this finding because in the FGDs, participants said things that suggested that they were not aware of the existence of these agencies, what they do and how they can be accessed.

*“There should be a Board that checks that the things being dispensed to people [by pharmacies] are proper*.*”* ***F*GD5**

*Participant (recounting an unpleasant experience at a pharmacy): I went back and was told that the person who’d sold me the medicine was not at work that day, even though they had the information in their computers. The problem wasn’t resolved yet I had given them my money*.

*FGD Moderator: Do you know where you can go to lodge a complaint if you’re unhappy with the way you’ve been treated at a pharmacy?*

*Participant*: *No*, *I don’t*. ***F*GD5**

#### Supply chain

Although supply chain issues were not touched on as frequently as others, a desire for price controls upstream the pharmaceutical value chain in order to mitigate the likelihood of unaffordable prices at the point of care, was expressed by one participant:

*“So*, *they should regulate prices right at [the state-owned pharmaceutical warehousing and distributing company]*. *If they don’t*, *then*, *the pharmacies will also charge us high prices*.*”* ***F*GD1.**

### Cross cutting factors: Market forces, equity, governance, donors and innovation

#### Market forces

Several business practices of private-sector pharmacists, (motivated by profit and/or market forces), were criticised by nearly all FGD participants. An example of such practices was the pharmacist-induced demand for medicines and testing services:

*“Do you know what pharmacy personnel do? They never fail to find something to give you even if it’s the wrong treatment.” **Text E in S1 Data***

*“We bought our own glucometers for home testing. If I go with [my own glucometer] to the pharmacy they don’t want to use ours because they want us to use theirs so that they can charge us a fee” **Text E in S1 Data***

Objectionable business practices by pharmacists also included charging a fee for conducting monitoring tests even in emergencies:

*Participant 1: “Pharmacies are where we get diabetes patients glucose levels monitored. Now we don’t understand because you might get charged 10 dollars today, the day after, 15 dollars and another day, 5 dollars. They charge us in U.S. Dollars. Blood pressure checking is what’s done for free but blood glucose testing, is not free.”*

*Participant 2: “That’s true, even if a diabetic patient is close to fainting, he can just faint; the pharmacy staff won’t budge, they won’t test him if no money is paid.” **Text E in S1 Data***

Other pharmacies rejected some health insurance schemes as valid payment and this too was considered unacceptable business practice,

*“Many pharmacies are rejecting health insurance.” **Text H in S1 Data***

The pricing of pharmaceutical services and commodities in foreign currency was also reported by participants as unacceptable

*“If we’re expected to pay in US dollars, it’s hard because we don’t earn them. It’s hard to move around searching for a different currency. They must just trade on terms that can be afforded by us Zimbabweans. If we have a local currency, let’s just use that.” **Text E in S1 Data***

Lastly, the placing of high profit mark-ups on diabetes medicines by pharmacies was also mentioned as an undesirable business practice.

*“let the profit not be something that can control you to the extent that you won’t see the human part to say these are humans also.” **Text A in S1 Data***

#### Equity

Frequently expressed was a serious dissatisfaction with the perceived inequity at policy level. This perceived inequity manifests as government’s apparent privileging of communicable diseases over diabetes, as exemplified by these quotes:

*“Diabetes is fatal so there should also be a diabetes tax (just as there is an AIDS Levy/tax)… Tuberculosis is treated for free so why is diabetes also not treated for free? So you see? We are just categorising them as similar conditions that are deadly. We must also be treated for free because I didn’t ask for this” **Text E in S1 Data***

Pleas were made for the government to consider that many diabetes patients may be vulnerable people who have limited means e.g. pensioners. This was followed by the suggestion that access to medicines for these vulnerable persons should not be associated with high prices:

*“In a way they should consider the population needing these medicines. Most of them are pensioners, they don’t have money. So if they can lower the prices of diabetes or stroke medicines and the like; all the crucial medicines, then also make them accessible at a public hospital like this one.” **Text B in S1 Data***

#### Governance

Participants had several ideas about the governance strategies that could be included in a national diabetes policy. These included: medicine price controls, regulation of the quality of both allopathic and complementary medicines accessible to patients, the judicious governance of national resources that contribute to government revenue (e.g. diamond reserves), a return to the policy of providing senior citizens access to healthcare free of charge, and the formulation of a sound monetary policy:

*“So*, *they should regulate prices right at [the state-owned pharmaceutical warehousing and distributing company]*. *If they don’t*, *then*, *the pharmacies will also charge us high prices*.*”* ***F*GD1**

*“I want to ask about herbal remedies peddlers. What do you think? They are saying if we use herbal remedies, our blood sugar levels drop…Some people are advertising those herbs even on broadcast media…is there a plan to safeguard us so that we won’t end up being sold these remedies because for sure they are cheap.” **Text F in S1 Data***

*“They should take the nation’s diamond proceeds and invest in hospitals. It would help. Instead of buying big cars at the expense of medicines. The money is there but they pocket it for themselves. The minister of health rides in an expensive car but the hospitals don’t have medicines”. **Text F in S1 Data***

*“What happened to that policy under which senior citizens like my mother there, got their medicines for free? Now we always buy.” **Text D in S1 Data***

*“The introduction of a local currency has caused confusion in this country. Quite honestly, we are very sore we are highly disadvantaged. I don’t know where to address it. Government or politics has injured us by introducing a new currency alongside the US dollar. When they introduced this currency, they said it was equal to the US Dollar but it is not the case. Some traders are only accepting US Dollars. If I don’t get US Dollars, then I don’t get medicines.” **Text H in S1 Data***

#### Donors

The perceived unaffordability of medicines and monitoring tests then led to the suggestions that a philanthropic entity be called upon to provide diabetes medicines for free:

*“We would like a donor to be found for us, who can supply us with diabetes medicines.” **FGD5.***

This apparent desire for donors to also prioritize diabetes medicines, may have been motivated by the observation many health products in Zimbabwe were ‘largely procured through donor funding’ [20].

#### Innovation

Regarding innovation, it was implicitly suggested that development of reusable glucose testing strips by be looked into:

*“I want to ask if there are types of sugar testing strips that can be wiped clean and reused? Because they are expensive. . .maybe I myself will invent something.” **Text F in S1 Data***

## Discussion

As expected, based on the extant literature, diabetes patients and their caregivers are interested in affordable medicines of good quality, accessed with minimum effort. Owing to the nature of the recruitment method (recruiting participants with a positive diabetes diagnosis, from major urban health institutions), it is not surprising that the views expressed by the discussants generally depict a patient population that is sensitive to the seriousness of diabetes, can articulate lived experiences with clarity and can link medicine access, a modified diet, monitoring tools and voice to positive outcomes. This is the kind of population able to provide clues about the contents of a developing country’s patient-centred-national diabetes policy. Most tenets uncovered from participant testimony relate to a recognition that diabetes is not simply an issue of pharmacotherapy but also of good governance, fairness, inclusiveness and humanity.

In this section, based on the results presented, we discuss our inductive interpretation of the ten pillars that the diabetes patients desire in a national diabetes policy. These pillars (discussed in turn below) are: financial protection, equity, a responsive workforce, consumer protection, allocative efficiency, price regulation, voice and representation, research and innovation, decentralised service delivery, and provisions for psychosocial support.

### Financial protection

Diabetes management, often being lifelong and multi-faceted, burdens households financially. Private health insurance in developing countries can cover some gaps in public spending [78]. However, Zimbabwean patients with health insurance are doubly disadvantaged when their health insurance plan is declined at the point of care. They pay out-of-pocket for medicines with no guarantee of a full reimbursement ex-post. They spend money on health insurance subscriptions and spend money again on out-of-pocket payments for medicines. Based on the aggrieved participants’ testimony it can be inferred that a sound diabetes policy ought to provide for the regulation of health insurance business practices. Predatory practices by health insurance providers and service providers doubly tax patients. Predatory practices by health insurance providers also discourage patients from purchasing health insurance.

### Equity

Noncommunicable diseases (NCDs) are catching up to infectious diseases as the leading cause of mortality in Africa [13]. This calls for NCD strategies that are as deliberate and effective as those designed for communicable diseases. In Zimbabwe, the privileging of communicable diseases (HIV/AIDS in particular) over NCDs is evidenced by several observations such as: the collection of an earmarked AIDS levy/tax, the existence of a dedicated statutory body dealing with HIV/AIDS and the availability of free medicines for HIV and Tuberculosis at public institutions. Furthermore, public institutions stock even the recently-developed medicines for third-line HIV treatment regimens. Whereas for diabetes, the public sector stocks and dispenses hypoglycemics developed as early as the beginning of the 20^th^ century. Frequently, interviewed participants referenced the prioritization of communicable disease management over NCDs justifying why diabetes deserves the same level of attention. A diabetes policy must be seen to be equitable and just.

### Responsive workforce

It has since been recommended that the number of health workers managing diabetes, as well as the level of diabetes knowledge they possess, should be increased [3]. Beyond this, the patients in our FGDs are additionally concerned with the health workers’ communication skills, business ethics and compassion. In a perfect market, patients are able to vote with their feet and seek medicines from pharmacies that meet desired standards of conduct. However, as the data suggests, patients seek medicines at outlets where prices are affordable or where their health insurance package is accepted, regardless of the quality of customer care there. A sound diabetes policy should have in-built governance safeguards that facilitate greater patient choice and enable patients to truly vote with their feet without being forced to give primacy to affordability over other dimensions of access.

### Consumer protection

In addition to financial protection, the protection of consumers of medicines is paramount. Patients lack the capacity to measure the quality of medicines at the point of care and may automatically trust pharmacies as sources of quality-verified medicines. In addition, consumers silently endure what they consider unethical business practices without recourse because of limited access to consumer protection agencies. A diabetes policy responsive to patient needs must provide for the empowerment of patients so that they may seek recourse when they feel they’ve been unfairly treated by the health system.

### Allocative efficiency

Patients who were deeply dissatisfied with the unavailability of diabetes medicines at the public hospital had ideas about the sustainable financing of medicines through earmarked taxation or the judicious use of revenue from Zimbabwe’s exportable natural resources. A strong diabetes strategy cannot escape the need for financial resources yet sub-Saharan health systems are generally inadequately funded to tackle diabetes [3]. From a patient’s perspective, corruption, bad macro-financial governance and lack of transparency about how funding for health is used, are certainly not the ingredients of a good diabetes strategy. Patients prefer that funding for health be visibly spent on items that they associate most with allocative efficiency, like medicines and rather than on items they associate with wastage, like vehicles.

### Price regulation

Although several policy options are available for low and middle-income countries who desire to control the price of pharmaceuticals [79], in Zimbabwe, the prices of pharmaceuticals are not regulated by the state through any codified framework [80]. This is thought to contribute to the variable and/or astronomical medicine prices. A diabetes policy that is acceptable to patients ought to pay attention to the prices of diabetes treatment consumables regardless of who pays for them.

### Voice and representation

The value of patient voice in matters concerning their treatment and on political forums has already been touched on in the introduction and was echoed by respondents. An advantage of including patient representatives at high levels of health system decision-making is that, patients gain a more informed understanding about how funding for health is gathered and used. A lack of transparency in this matter heightens suspicions of corruption and mismanagement.

### Research and innovation

Reusable glucose test strips were not in existence at the time of the study. However, the observation that some FGD participants suggested that reusable glucose test strips should be invented, taken together with the frequently-expressed theme of expensive glucose monitoring, shows a desire for an innovation-oriented approach to diabetes policy formulation.

### Decentralised service delivery

A fair diabetes policy ought to be cognisant of the inconveniences associated with requiring diabetes patients to regularly travel from different regions of the country to attend routine outpatient clinics at the largest referral hospital in the country. This tended to occur for several reasons. First, the user fees at lower levels of care were higher. Second, the medicines patients took were not classified as those that can be stored or dispensed at lower levels of care. Third, the referral hospital in the capital city was where diabetologists could be accessed. Decentralization of diabetes management to lower levels of care has been done with some success elsewhere in Africa [81].

### Psychosocial support

A reliance on psychosocial networks by persons living with or affected by diabetes, was evident [see IHC Quotes]. Support groups for diabetic patients have been shown to improve clinical outcomes [82]. Diabetes patients and their caregivers already congregate regularly at outpatient clinics for monitoring and prescription renewal. Building on this to form structured local support groups and social ties may improve patients’ collective voice, chances for collaboration on income-generating projects [83] and mental health [84]. Formal inclusion of community support groups within a diabetes policy framework would increase their legitimacy and possibly widen their access to funding as a consequence.

### Future research direction for a comprehensive diabetes policy

Our work in this paper documents the qualitative research conducted with people living with diabetes. While their lived experiences constitute invaluable input into any national diabetes policy, it is worth highlighting that further research with other stakeholders, utilising quantitative and mixed methods approaches is also necessary. These types of research could include: cost-effectiveness studies and other economic analyses to support the selection of one policy option over others; epidemiological studies focusing on patterns of morbidity and mortality; pharmacological studies and clinical trials to test and validate on new treatment modalities for diabetes patients.

#### Conclusion

As SSA countries prepare to devise and operationalise national diabetes policies, most of which are rightly concerned with the prevention, diagnosis and treatment of diabetes and its complications, it is important to attend to the interests of those with lived experiences of diabetes. We set out to discover some of these interests by qualitatively capturing the challenges and suggestions by a population of diabetes patients in Harare, Zimbabwe. We concluded that in addition to the already documented provisions for access to affordable and universal prevention, diagnosis and treatment modalities, a diabetes policy acceptable to patients is one infused with principles of good governance, fairness, inclusiveness and humanity. Such principles manifest as provisions for: financial protection and price regulation, consumer protection, equity in the attention accorded to different diseases, decentralized service delivery, inclusion of patient voice in political decision-making for a responsive compassionate health workforce, psychosocial support for patients and their care givers and allocative efficiency and transparency in public expenditure.

## Supporting information

S1 Table Qualitative data coding framework. (DOCX)

S2 Table Selected quotes from the data. (DOCX)

S1 File FGD guide (English). (DOCX)

S2 File FGD guide (ChiShona). (DOCX)

S1 Data FGD transcripts. (DOCX)
