# Supplementary material for: Perceptions of diabetes patients and their caregivers regarding access to medicine in a severely constrained health system: A qualitative study in Harare, Zimbabwe
Source: PLOS Glob Public Health. 2022 Mar 3;2(3):e0000255. doi: 10.1371/journal.pgph.0000255 (PMC10021663; doi:10.1371/journal.pgph.0000255)
Supplement: S1 Table — (DOCX) [file pgph.0000255.s001.docx]

# **S1 Table: Coding Framework**

Table 1: Qualitative data Coding Framework

| Code/Subcode | Description of Code | Code data at this code if it mentions: |
| --- | --- | --- |
| Individual, household and community | Socio-cultural practices and beliefs which influence decisions about access [9] | Things people think or believe about illness, healthcare, health facilities, medicines, money, family, etc. that influence if, when and how they decide to access medicines. |
| Availability | The relationship between the type and quantity of product or service needed, and the type and quantity of product or service provided. Is it there where it is supposed to be? [13] | The presence (or absence) of medicines at places that they are supposed to be |
| Affordability | The relationship between prices of the products or services and the user’s ability to pay for them. Can patients get it without incurring financial hardship? [13] | Pricing issues, or the unaffordability of medicines or costs related to acquiring medicines (includes cost of transportation, tests, or Doctor consultation) |
| Acceptability | The relationship between the user’s attitudes and expectations about the products and services and the actual characteristics of products and services. Is the patient happy with the medicine (packaging, dosage form, colour, smell, the provider’s characteristics or other aspects of the medicine or service etc?) [13] | Issues/complaints to do with how the medicine or the service provider is. E.g. complaints about the inconveniences of using needles and syringes for insulin injection instead of pen-sets or the demeanour of the pharmacist |
| Accessibility | The relationship between the location of the product or service and the location of the eventual user of the product or service. Can the patient reach the place where the medicine is with reasonable effort? [13] | Issues to do with the ease of accessing the health facility where the medicine is, such as distances travelled. |
| Quality | The fitness of the medicine for human consumption adherent to regulatory standards [15 | Issues to do with the quality assurance of medicines available to patients. |
| Equity | Fairness and justice in access. Where those disadvantaged in any one way, are assisted to achieve access like those that are not similarly disadvantaged (55) | Issues to do with fairness or unfairness,  Issues to do with helping the poor or disadvantaged |
| Governance | Stewardship and oversight over all actors in a health/pharmaceutical system. This includes oversight over government itself, manifesting as public accountability, freedom from corruption and bureaucracy [9] | Issues to with the government’s regulatory role in all aspects of access.  Issues to do with corruption, accountability of actors including government. |
| Transparency | The availability of information about price, source, availability, or quality of medicines [9] | Issues to do with information access and ease of information access. This includes information about price, availability or quality of medicines. It also includes transparency of healthcare decisions made by providers (do providers explain care decisions to patients for instance) |
| Innovation | The ingenious development/discovery/invention of strategies or interventions or products to improve access to medicines [9] | Issues to do with strategies, ideas, products, etc. which improve access to medicines |
| Donors’ Agenda | Donor activities that influence plans, policies and practices [9] | Anything to do with donor activity |
| Market forces | Pluralism and competition of service providers and the consequences of this pluralism and competition [9] | The effects of competition and pluralism of pharmaceutical service providers |
| Health Financing | Issues to do with the pooling and deployment of funds in the pharmaceutical system and the financial protection of households [55] | Anything to do with health insurance, out-of-pocket expenditure or public expenditure for health |
| Human Resources | Health workers interfacing with diabetes patients including prescribers and pharmacy personnel [55] | Anything to do with participants’ perceptions of health workers |
| Health Information | Involves the management of information that enables decision-making at all levels of the health system [55] | Anything to do with the provision of information or lack thereof |
| Supply Chain | The channel that delivers quality and cost-effective medicines and health technologies from their source to the patient at the point of care [55] | Anything that touches on any aspect of the supply chain. |
| Service Delivery | The delivery of quality health interventions (including preventative, diagnostic and curative ones) [55] | Anything to do with how participants access services |
| Other | Any code that is not explicitly described in this table | Anything that cannot be coded anywhere else but is determinant of access. |
