# Supplementary material for: Perceptions of diabetes patients and their caregivers regarding access to medicine in a severely constrained health system: A qualitative study in Harare, Zimbabwe
Source: PLOS Glob Public Health. 2022 Mar 3;2(3):e0000255. doi: 10.1371/journal.pgph.0000255 (PMC10021663; doi:10.1371/journal.pgph.0000255)
Supplement: S2 Table — (DOCX) [file pgph.0000255.s002.docx]

# **S2 Table. Supporting quotes from qualitative data.**

Supporting quotes from the data relating to individual, household and community factors

| *“Because of our diabetes, we are close to death. We can die anytime. The life of a person with AIDS is actually different from ours”.* ***Text F in S1 Data*** *[IHC1]* |
| --- |
| *“I wasn’t taking diabetes medication for 5 days therefore my blood sugar levels rose”.* ***Text B in S1 Data*** [IHC2] |
| *“You get to a pharmacy and you’re that metformin has run out, only glibenclamide is available. I then buy what’s there and take what’s there, neglecting the fact that the medicines are a course that must be taken together”.* ***Text H in S1 Data*** *[IHC3]* |
| *“If I find sugar expensive in a supermarket, I look for it from the street vendors outside; they might be cheaper. But for medicines, it’s a problem. You cannot buy medicines from street vendors”.* ***Text A in S1 Data*** *[IHC 4]* |
| *“If you care for a diabetic and hypertensive patient, you need to know always know. You know, BP is different from diabetes. I am hypertensive- when I’m sitting, I can feel it when my BP is rising. But for a diabetic, you might be conversing with them without knowing that while you’re laughing their blood sugar is rising such that you suddenly find yourself talking to yourself. So, people like that are not supposed to be denied tests even when they don’t have money. What’s more important, money or human life? Jut testing only/ Not dispensing a tablet but just testing only”*  ***Text E in S1 Data*** *[IHC5]* |
| *“We don’t have the food they need to eat…”* ***Text C in S1 Data*** *[IHC6]* |
| *“But why are we falling short? Our patron is the deputy minister surely… our patron being a diabetologist and a deputy minister…I was happy when he was appointed deputy minister, thinking now our problem is resolved… But we seem to be getting worse.”*  *“Those people don’t listen!”*  ***Text H in S1 Data*** *[IHC7]* |
| *“This particular medicine is very expensive….So here [at the public hospital], you won’t find it. You might find Enalapril, Nifedipine, HCT and metformin but the expensive ones, it’s hard. Such that at the end of the month you become stressed truly, I end up developing high blood pressure too”.* ***Text D in S1 Data***  *[IHC8]* |
| *“You just sacrifice in order to buy the medicine but you won’t be having the money. Like myself, I don’t work, but I’m my mother’s caregiver. I am someone else’s dependent. So I then ask that someone else, for money to buy medicines for my mother”.* ***Text A in S1 Data***  *[IHC9]* |
| *“One of the challenges is that, if you tell a diabetic family member that there’s no money they don’t understand.”*  *“If you then go on to buy something else, they ask, ‘So there’s no money, huh?’”* ***Text C in S1 Data*** *[IHC10]* |
