# Supplementary material for: Perceptions of diabetes patients and their caregivers regarding access to medicine in a severely constrained health system: A qualitative study in Harare, Zimbabwe
Source: PLOS Glob Public Health. 2022 Mar 3;2(3):e0000255. doi: 10.1371/journal.pgph.0000255 (PMC10021663; doi:10.1371/journal.pgph.0000255)
Supplement: S1 File — (DOCX) [file pgph.0000255.s003.docx]

**S1 FGD Guide (English)**

General

1. I would like to find out where you find diabetes medicines when you receive a prescription. What do you do? Where do you start looking?

Availability

1. Do you find your medicines here at the public hospital pharmacy?
2. What do you do when you don’t find them?

Affordability

1. When you say the medicines are expensive, do you mean in the local currency or in US Dollars?
2. Are they expensive but available or both expensive and unavailable?
3. Do you think the prices of medicines being charged at pharmacies are justified?
4. What if the places where pharmacies order medicines are also expensive?
5. Someone might say, ‘I’m in the private sector. I’m meant to make a profit. It’s the public sector that’s supposed to be dispensing low cost medicines.’ What do you say about that?
6. We hear people calling for the reduction of medicine prices at private pharmacies but not the same for other commodities like sugar. What is unique about medicines?
7. When you’ve been given a price quote in USD, from where do you acquire the US dollars?
8. What’s going on with Health insurance?
9. Are you on any health insurance plan?
10. Would you join a health insurance scheme if one is suggested to you?

Accessibility

1. Have you ever asked the community Pharmacist to ask around on your behalf if a different pharmacy has your medicines in stock?
2. Do you move around multiple places or do you buy at the first pharmacy that you find them?
3. When you buy medicines at private pharmacies, do you find them at the first pharmacy you look?

Acceptability

1. If you find a cheap Pharmacy, is this where you buy irrespective of how you’re treated there?

Advocacy/Voice

1. Have you ever organized yourselves as diabetes patients to lobby the government as a group?
2. Do you know about the Zimbabwe Diabetes Association?
3. Do you know where you could report if you’re treated badly at a pharmacy?
4. Do you know where you could report when you have been sold expired medicines?

Quality

1. Have you ever been concerned about the quality of the medicines you get from pharmacies?

Information

1. If you visit the Pharmacy or the doctor, are you given medicine information such as information on side effects or adverse reactions?
2. Would you like this information?
3. Would it help you to have a way of comparing medicine prices remotely from home?

Other

1. Are there any other issues that you encounter during your search for medicines, apart from affordability issues?
2. Is there any other suggestion about potential solutions you wish policymakers would heed?
