# Supplementary material for: Perceptions of diabetes patients and their caregivers regarding access to medicine in a severely constrained health system: A qualitative study in Harare, Zimbabwe
Source: PLOS Glob Public Health. 2022 Mar 3;2(3):e0000255. doi: 10.1371/journal.pgph.0000255 (PMC10021663; doi:10.1371/journal.pgph.0000255)
Supplement: S2 File — (DOCX) [file pgph.0000255.s004.docx]

# **S2 FGD Guide**

General

1. Ndinoda kuziva kwamunowana mishonga yeShuga paya pamunenge manyorerwa mishonga iyi naChiremba. Munodini? Munotangirepi kutsvaga?

Availability

1. Munowana mishonga yenyu pano pachipatara chehurumende here?
2. Kana musina kuuwana pano munozoiwana kupi?

Affordability

1. Kana muchiti mishonga yenyu iri kudhura, munoreva nemari yemuno here kana kuti nemari yekunze kwenyika?
2. Mishonga iri kudhura ichiwanikwa here kana kuti iri kudhuru zvekare ichishaikwa?
3. Munofunga kuti mitengo yemushonga kuzvitoro zvinotengesa mishonga, yakakodzera here?
4. Ko kana kuya kunohodhwa mishonga nevanozokutengeserai kuchitodhurawo?
5. Umwe munhu angati, ‘Ini ndiri muzvinabhizimisi, ndinotofanira kuwana mari inobva mubhizimisi iri. Kuzvipatara zvehurumende ndiko kunofanira kutengeswa mishongayakachipa, kwete muchitoro mangu.’ Munoti kudii nechirevo ichi?
6. Tinonzwa vazhinji vachiti mitengo yemishonga ngaideredzwe kuzvitoro zvinotengesa mishonga asi hatinzwe zvimwechetezvo pamusoro pezvimwe zvinhu zvinotengeswawo nemamwe mabhizimisi zvinenge tsvigiri. Chii chakanyanyokosha pamushonga?
7. Kana maudzwa mutengo wemushonga nemari yekunze kwenyika, munoiwanepi mari yekunze kwenyika?
8. Chii chiri kumboitika nemaMedical aid?
9. Muripo here pachirongwa cheMedical aid?
10. Mungazviisa here pachirongwa cheMedical aid kana mukaziviswa nezveimwe yadzo?

Accessibility

1. Makambokumbira here muzvinachitoro chemishonga kuti akubvunzirei kune vamwe vake kuti mishonga yenyu ingawanikwa here kune zvimwe zvitoro zvemishonga?
2. Munofambafamba muchitsvaga mishonga yenyu kuzvitoro zvakasiyanasiyana here kana kuti munotenga muchitoro chamunotanga kuwana mushonga yamuri kutsvaha?
3. Kana muchitenga mishonga muzvitoro zvemishonga, munoiwana muchitoro chamunotanga kupinda here?

Acceptability

1. Kana mawana chitoro chine mishonga yakaderera mutengo, ndimo mamunotenga here kunyange musiri kubatwa zvakanaka imomo?

Advocacy/Voice

1. Makambozama here kubatana sevanhu vanorarama nechirwere cheshuga, kuti mukumbire hurumende rubatsiro pazvichemo zvenyu?
2. Munoziva here nezvegungano revanhu vanorarama nechirwere cheshuga rinonzi Zimbabwe Diabetes Association?
3. Munoziva here kwamunomhanh’ara kana muchinge mabatwa zvisingakufadziyi muchitoro chemushonga?
4. Munoziva here kwamunomhanh’ara kana muchinge matengeserwa mishonga yadaridza zuva rainofanirwa kumwiwa?

Quality

1. Makambonetsekana here nezvemhando yemushonga yamunotengeserwa muzvitoro zvemishonga?

Information

1. Pamunoenda kuchitoro chemushonga kana kuti kwaChiremba, munopiwa here ruzivo pamusoro pezvinhu zvinogona kana umwe urwere hunogona kukonzerwa mumuviri wenyu nemishonga yeshuga yamuri kumwa?
2. Mungada here ruzivo urwu?
3. Zvingakubatsirai here kuwana nzira yekuenzanisa nayo mitengo yemushonga muzvitoro zvakasiyanasiyana, muri kumba kwenyu?

Other

1. Pane zvimwe here zvamunosangana nazvo pakutsvaga mishonga yeshuga, zvamunoda kukurukura nezvazvo, zvisinei nemitengo?
2. Mune mazano amunawo here amunoda kuti vatungamiriri venyika vatevedzere mukugadzirisa zvinonetsa?
