# Supplementary material for: Perceptions of diabetes patients and their caregivers regarding access to medicine in a severely constrained health system: A qualitative study in Harare, Zimbabwe
Source: PLOS Glob Public Health. 2022 Mar 3;2(3):e0000255. doi: 10.1371/journal.pgph.0000255 (PMC10021663; doi:10.1371/journal.pgph.0000255)
Supplement: S1 Data — (DOCX) [file pgph.0000255.s005.docx]

**S1 Data. Transcripts**

**Text A**

Interview text

I: Sakaa nhasi tIri kukurukura pamusoro pee pemushonga ye diabetes.

All R: Yes.

I: Eee munogona kushandisa chirungu kana Shona whatever language yakakusunungukirai.

All R: Uhmm.

I: Eehee ini ndaona kuti munoperekedza vanorwara.

R1: Uhmm.

I: Imimi matouya kuzoitawo review.

R2 Uhmm.

I: Ndaona muchifara chaizvo nemaresults enyu.

R2: Yes.

I: Iii ndahwa muchiti mavane makore maviri.

R2: Ehe

I: imi maiti mai vakabatwa three months, two months ago?

R1: Two months ago ehe.

I: Saka ndinoda kunzwisisa kunyanya nyanya kuti mishonga ye shuga munoiwana kupi kana mainyorerwa. Masvika pano manyorerwa mushonga yeshuga. Munodii? Munotanga kutarisa kupi?

R1: Uhmm inini zvandakaita ndakanyorerwa mushonga ka pakutanga ndobva vati endai munotenga ku pharmacy. Saka ndakatanga kutarisa pharmacy yepanaapa ndobva ndaushaya, ndobva ndazoenda ku pharmacy yemu town. Inonziii Word, Word chii handichanyatsozivi ndooyandakazotenga. Pavakazobuda futi vabva vandinyorera futi mumwe mushonga wekuzvibaya. Vaakuzvibaya. ndobva ndanzi ndonotenga futi ku pharmacy ndobva ndanotenga futi kupharmacy 6 US nema syringe acho.

I: Uhmm.

R1: Ndobva ndanotenga.

I: Uhmm.

R1: Ehe ndobva ndanotenga.

I: US makatobhadhara ari ma US chaiwo?

R1: Ehe ndakabhadhara 6 dollars US.

I: Koo imimi munozvifambisa sei?

R2: Inini ndiri medical aid saka vanonditi shortfall pama pen sets.

I: Uhmm.

R2: Saka ndakatoona kuti pen set yacho vaidaa about 34 bond.

I: Uhmmm.

R2: Saka ndikati handina saka ndava kutongo dependa neee vial yekuzvibaya iya ye syringe iya yakati cheapeyi but inenge ine shortfall ye about 6 dollars.

I: Pen set yakanakirei?

R2: Pen set yakanakira kuti haindinetsi handinetsekani paku adjuasta ma millimetres acho andinofanirwa kuisa because ndino ngotuna yosvika pandiri kuda ndobva ndangobaya.

I: Uhmmmm.

R2: Manje iyi you find kuti chii, syringe kuti unatso adjusta kuti unatsosvika measurement yacho at times anongona at times kunge ari manheru, globe rinenge risinganatsooni zvakanaka saka uno but unenge wakutongofunga kuti ndasvika approximately pamamilliliters andiri kuda.

I: Alright.

R2: Yes saka ndiyo disadvantage yayo pa measurement apo.

I: Asi insulin ichiri kuwanika zvayo nema bond.

R2: Aaa insulin nema bond so far iri kuwanika but issue ye shortfall iyoyo ndoo ine nyaya

I: Uhmm.

R2:Zvekuti dai zviri kunzi waikwanisa kuitawo something vakatii… kunonzi kuita sei.

I: Ehe.

R2: Kuti subsidisa

I: Uhmm.

R2: Kuti tiwane mishonga iyoyo pasina shortfall iri kutaurwa.

I: Pamakazowana mushonga ku Word Pharmarcy.

R1: Uhmm.

I: Makangobva panaapa ndobva makananga ku Word straight?

R1: Uhmm umm ndakambotenderera ndichiushaya.

I: Waitoshayika kana kuti waidhura?

R1: Kwanadakatanga kuona kwawaidhura ku Olive iri pa Montagu iyi.

I: Uhmm.

R1: Koita pa Baines panga pasina. Ndobva ndaenda pa Trinity waidhura futi saka ndanga ndakungofamba ndichitsvaga kuti pakacheaper ndepapi. Pandakazoiwona mu Word imomo ndomandakaiona ichiita 6 dollars.

I: Uhmm munombofamba famba muchitsvagawo here kana kuti munongoti pamasvikira mukaiwana ndipapo?

R2: Inini ndinongoti panotorwa medical aid yangu ndipo pandinoenda.

I: Hoo?

R2: Handitomboenda ku cash because handina cash yacho.

I: Hoo horaiti.

R2: Ndinoenda ma pharmacy angu andinoziva kuti ndiwo anotora medical aid yangu.

I: Hoo motoaziva ma pharmacy acho anotora medical aid?

R2: Ehe ndotomaziva.

I: Aaa horaiti saka munoshandisa whattsapp here pa phone penyu?

R1: Ehe.

I: Koo imi munoshandisa whatsapp here?

R2:Ehe ndinoshandisa.

I: Munoziva here kuti pane group rekuti kana ukabvunza kuti mushonga wakati uri kuwanika kupi inokuudza?

All R: Hatirizii.

I: Koo pharmacist, mukapinda mupharmacy mukati muri kutsvaga zvamuri kutsvaga vakati Havana, vanombo offerwo here kukubatsirai?

R1: Uhmmm.

I: Kuti regai ndikubvunzirei kwazviri?

R1: Uhmm kwandakaenda havana.

I: Vanongoti hatina?

R1: Uhmmm.

I: Munobvunza ani we pa front paya paya?

R1: Mu pharmacist wacho.

I: Munotobvunza ma pharmacists ari kuseri kuya?

R1: Hanti ndiye anenge ari panenge paine computer apa?

I: Ehe.

R1: Ndowandinovhunza ka iyeye.

I: Pane computer ka pane vamwe vanenge vari ipapo.

R2: Masecretary.

I: Pane pa till pane computer ka pa OTC paye.

R1: Pane panenge pakatonyorwa kuti prescription [overtalk] chii chii ndiko kwandinoenda.

I: Ehee ndookwamunoenda vongoti hatina votokusiyayi muchienda?

R1: Seni hapana munhu akambonditi endai kwakati

I: Nekuti ma pharmacist ane ma group avari.

R1: Uhmm.

I: Vamwe vanobvunza pa group kuti pane patient yangu iri kutsvaga chakati pane anacho here.

R1: Uhmm.

I: Then wotonzwa mumwe munhu achiti ngaauye ku pharmacy kwangu then obva akuudza kuti ndanzwa pa phone pangu pari kunzi chii.

R1: Uhmmm.

I: Saka vazhinji vanogona kukubatsirai saizvozvo kana vasiri busy I suppose.

R2: Ini ndaisaziva kuti pane facility iyoyo.

I: Ehe mukapinda mu pharmacy mukashaya zvamunoda munogona kubvunza pharmacist iyeye kuti hauna here ma group ako anunogona kundibvunzira .

R2:Hoo ok.

R1: Horaiti.

I: Nekuti kana asina kubvunzwa dzimwe nguva anongoti aa handina ongokusiyai muchienda but mukamuratidza kuti munoziva kuti ane ma whatsapp group anoombopinda anotaurwa zvinhu zvakadaro anogona kuti aaa regai ndikubvunzirei mombomira 5 minutes.

R1: Horaiti.

I: Achibvunza pa group. Saka matambudziko…. besides kuti zviri kushaikwa munofanira ku tenderera tenderera.

R1: Uhmm.

I: Kana kuti kana mauwana unochargwa nema US munawo here ma US munomawana kupi?

R1: Hatimbomawana unotozongo sacrifisa kuti tiatsvage.

I: Uhmmm.

R1: Unotozongo sacrifisawo kuti rega nditenge but unenge usina.

I: Hoo ok.

R1: Seni handiende kubasa.

I:Uhmm.

R1:Ndini ndinochengeta mhamha.

I: Uhmm.

R1: Ndotochengetwawo nemumwe munhu.

I: Uhmm.

R1: Saka munhu uyu ndiye wandinotokumbirawo kuti itondipaiwo mari yekutengera mhamha mushonga.

I: Uhmm havasi pamedical aid mhamha?

R1: Havasi.

I: Alright ok. Kuri kuti pawanikwa medical aid mungaijoina?

R1: Uhmm mari yacho so far handina.

I: Horaiti.

R1: Mari yacho so far handina.

R2: Medical aid ishomaka as compared to yamunozoburitsa.

R1: Uhmm.

R2: Ye medical aid ishoma. You find hakuna hamumbobatirwa more than 10 dollars I suggest muto joina medical aid kutaura chokwadi.

R1: Humm.

R2: Because inini pandakazonzi shortfall iya iya its only that medical aid ndoo inenge ichi cover. Vanozonditi mushonga yepapa vachizonditi shortfall ye 6 dollars zvese ma pen set mushonga yacho nejekiseni.

R1: Horaiti.

R2: Zvinenge ndaakuzongobhadhara 6 dollars. Zvitori nani kubhadhara 6 dollars iyoyo pane kuti ma US amuri kutaura rate izvozvi imarii. Saka munotozoona kuti mukabhadhara medical aid munenge mava kuto saver than 6 dollars every month.

R1: Uhmm.

R2: Hanti mazviona.

I: Eee munombotanga mabvunza kuti medical aid inoita zvevanhu ve diabetes ndeyipi munozojoina isingatengi insulin.

R1: Horaiti.

R2: Ok.

I: Ehe munofanira kutanga ma…pane vanenge vachitoziikanwa senge Premier.

R2: Ehe Primier.

I: Ehee nedzimwewo munongobvunza kuti ma members enyu e diabetes munozvifambisa sei.

R2: Ehe.

I: Motsvaga the best medical aid for diabetes I think that would be the best. Uhmmm ndanzwa muchiti mune metformin futi.

R2: Ehe.

I: Munomboita here worry kana concern yekuti mushonga yamuri kutenga metformin yamuri kutenga imetformin yechokwadi?

R2: Haa ipapo handisati ndamboita worry because pharmacy yandinotenga iri reputable saka assumption ndeyekuti vanotengesa zvinenge zviri chaizvo chaizvo.

I: Uhmm.

R2: To be honest handisati ndambozvii.

I: Hamusati mamboita worry.

R2: Ehe.

I: Kana kumboita complaint kuti iri kufufunyuka kana kuti kana.

R2: Handisati but ndakazombonzwa last week iyo vanhu vachitaura kuti, shamwari yangu yaitongondiudzawo kuti vanhu vari muma pharmacy vari kuti mishonga inenge ya expire but in fact havasi vemapharmacy as such. Vepharmacy vanotora mishonga yavo voiyendesa mu bin.

I: Uhmm.

R2: Pane vanozotora manje mu bin imomo vonoitswanya tswanya...

R1: Vonoitengesa.

R2: Aiwa vanoitswanya tswanya voiita repack, because vakaitengesa yakadaro inenge ichitooneka kuti expiry date ndi nezuro.

I: Hoo.

R2: Saka vanoitora manje vonoipwanya pwanya votsvaga tuma container voita refill. imimi munenge muchitofungidzira kuti pamwe igenuine mushonga iyo isiriyo asi. To answer your question handisati ndambozvitarisa zvangu kuti I genuine here kana kuti haisi genuine.

I: Ok saka hamusati mamboita henyu concern iyoyo?

R2:Aaa handisati.

I: Hooo horatit hoo its fine saka mungati pane mamwe ma issues here amunowanzosangana nawo pamunotsvaga mushonga besides kuti iri kudhura inoda shortfall kana kutiii yaa either way kuti ndoo dambudziko ramataura iroro rekuti..?

R2: Aaa handifungi kuti pane mamwe ma issues because kungoti zvayo mishonga yacho yanga ichiwanika zvekuti ndikashaya mu pharmacy iyi ndoenda kune iyo. handizofi ndakaishaya pama pharmarcy ese anotora medical aid. Handisati ndamboishaya.

I: Oh alright that’s alright zvakanaka chaizvo. I think ndoozvega kana pane zvimwe besides ku subsidiswa?

R2: Uhmm.

I: Pane imwe solution yamunga suggesta kuti dai vamboteererawo izvi?

R2: Haa iyoyo yekuti dai vatipawo mishonga mahara iyo I think zvingatibatsire.

I: Uhmmm.

R2: Haa because imagine 6 dollars iri kutaurwa apo inobva kupi 6 dollars yacho?

I: Uhmm.

R2: unenge uchitambiraweo ka 400 dollars pachada rent pachada chii saka you find kuti hatizokwanisi kuwana mari yekutenga mishonga iya iya.

I: Uhmmm.

R2:Zvekuti eventually vanhu vachazopedzisira vasingachakwanisi kuitenga mishonga yacho.

I: Uhmm.

R2: Kana Zvikaramba zvakaita zvazviri kuita izvi.

I: Asi munofunga kuti mitengo iri ku chargiwa iri justified?

R2: Haaa ini handifungi kuti iri justified ini because haaa ndisingazivewo hangu but ndinoona kunge mari yacho yakawanda iri ku chargwa yakawandisa zvisingaite.

I: Yakawandisa?

R2: Yakawandisa ehe .

I: Koo kana kwavanozvitenga zvichidhurawo?

R2: Handizozivawo because handiti zvinonzi inouya yoendeswa ku Natpharm here?

I: Ehe.

R2: Saka ku NATPHARM ikoko ndokwawanofanira kubva vagadzirisa ma price acho.

I: Ehe ehe.

R2: Saka vakatiregerera zvichidhura ikoko vemumapharmacy vanozongotidhurisirawo.

I: Ehe.

R2:Tisingazoziva kuti vanenge vava kuita profiteering here kana kuti vanenge vachingoisawo ka markup kashoma vari kuitengawo ichidhura.

I: But mumwe munhu anogona kuti nekuti I private sector basa rayo nderekuita profit making.

R2: Uhmm.

I: Ndozvavakavhurira ma door, public sector ndoo inofanira kunge ichipa vanhu mishonga yaka subsidiswa.

R2: Uhmm.

I: Munoti chii nazvo? vanoti ehe kana ndiri kuita profit ndoozvandakatovhurira ma door nekuti ndiri mu private sector?

R2: Aaa but munhu anouya mu pharmacy haazoti ndiri private kunongouya munhu wese saka I don’t think akatora munyati iwoyo kuti ndiri private, ndofanirwa kuita… anofanirwa kunzwirawo tsitsi patient uyu auya kuzotenga mushonga.

I: Uhmm zvakasiyanei nekutenga kana shuga mu shop nekuti hatisati tambonzwa OK ichinzi ngainzwirewo tsitsi itengese shuga nemutengo uri right?

R2: OK manje muenzaniso wamapa ndewekuti OK manje ndikaona shuga ichidhura ndinonotsvaga panze pane vanotengesa panze vanogona kunge vakacheaper, but mishonga manje inonetsa kuti handinganotenga mushonga panze. Haiwanike.

I: Aaaa haaa.

R2:Handiti mazviona saka ndoo pandiri kuti ndoo pane challenge. Ma OK ndogona kunozvisiya ndichiti zviri kudhura ndoenda kunotsvaga panze paya.

I: Eheee.

R2: Manje mushonga there is no two ways kutodzokera ku pharmacy ikoko.

I: Ehe aiwa ndanzwisisa explanation yenyu tatopedza soo.

R1: Mainini vangu hava kuziva kuti ndatorwa bp. [side-talk, nothing to do with the research matter]

I: Haa ok saka ndazosara ndichibvunza kuti besides kuti mishonga inombodhura inombonetsa kuwana munoita muchifamba muchifamba.

R1: Uhmm.

I: Munomboita here worry yekuti mishonga yamuri kuwana haisi quality svinu?

R1: Inini handisati ndambozviziva kuti kunowanika ma fake.

I: Uhmm.

R1: Semunhu anenge achitsvaga muma pharmacy saka ndingotrusta pharmacy kuti ku pharmarcy ndinokwanisa kuwana mapiritsi akaita sei akanaka.

I: Uhmm ok. saka ndeapi ma solution amunofunga kuti anogona kukubetserai pane matambudziko amunosangana nawo kana muchitsvagira mai mushonga?

R1: Dai mimwe mishonga ichiwanika pa chipatara zvekuti ukatengera pachipatara zvinoti cheapeyi than kunotsvaga kuma pharmacy.

I: Uhmm saka munofunga kuti mamwe ma price ari kuchargwa nema pharmacy akakodzera here kana kuti vari kutowedzera zvavo ma price?

R1: Vari kudhura zvavo. Fanika nyaya yekunzi ma US apa.

I: Nekuti ivo vanoti mishonga yatinotengesa tinoIwana kunze, kunze hakutambirwe ma bond. Tinotenga nema US saka ndoosaka tichikwidza mutengo.

R1: Plus zvavari kuzoita ndezvekuti vanogona kuti drug ravari kuti 5 dollars bond.

I: Uhmm.

R1: Vobva vati dollar US saka inini pandichawana dollar iri ndichida kunoritengesa kuti ndizotenge ne bond ndoritengesa nemarii? saka unozongoona kuti kutenga ne US kwacho kutori nani than kutenga ne bond.

I: Hooo saka better ku…

R1: Kutongonotenga ne US racho because ndikada kutenga dollar ne US ndogona kutenga 250.

I: Hoo saka muri kuti rate yemu pharmacy ne rate yemu road zvakasiyana? [overtalk]

R1: Ehe eee ndozvavari kuita.

I: Haa horaiti.

R1: Zvakafanana ne metformin iyoyo.

I: Uhmm.

R1: Inini ndakaitengaaa ndakaitenga marii ko inini umm ndakukanganwa price yayo yandakapedzisira kutenga nayo.

I: Its ok its alright.

R1: But kuitenga ne bond yaidhura than kutenga nechii ne US.

I: Ok.

R1: Uhmm.

I: Ok saka unenge wava ku calculater kuti saka ndochinja here kana kuti…

R1: Ndotongotsvaga hangu munhu ari kutengesa US ndotora US ndonotenga than kutenga ne bond.

I: Uhmm. Saka tanzwisisa kuti ma pharmacy ari kudhura ma private pharmacy munofunga kuti vanofanirwa kudhurisa here kana kuti nekuti kana vari kuti ivo kwavari kuzvitenga zviri kudhurawo munoona vane kodzero here kuti vadhurisewo?

R1: Aiwa ehe chinhu chese chaunotengesa ka.

I: Ehe.

R1: Unotengesa zvichienderana nekuhorda kwawakaita.

I: Ehe ndizvozvo.

R1: Saka hapana chatingakwanise kuchinja because ndoozviripo.

I: Uhmm. Pane zvimwe zvamanga muchida kutaura wo here zvinoenderana nemushonga chero chamunofunga kuti chinogona kukubetserai?

R1: Haa handina.

I: Tatopedza saka.

R1: [Laughing] horaiti.

**Text B**

I: Saka kuti tingotangisa iiii ndakukokai kutsvakiridzo ku research yangu nekuti ndinoda kuziva matambudziko amunosangana nawo kana muchitsvaga mushonga wenyu kana kuti muchitsvaga mushonga wevamunoriritira vane chirwere cheshuga.

R: Uhmm.

I: Saka ndinoda kutanga nekubvunza kuti kana manyorerwa prescription pano munodii?

R1: Tinoenda paa pharmacy apo.

I: Uhmm.

R1: Kana vainawo vanotipa.

I: Uhmm.

R1: Yavasina tava kuenda kune mamwe ma pharmacy tonotsvaga. Dzimwe nguva mari ndiyo inokurira.

I: Is it the same nemi?

R2: Yaa it’s the same uhmmm it’s the same process yataurwa na mhamha.

I: Uhmm.

R2: Then tozotanga kutsvaga ma other pharmacies but big challenge inyaya ye I think accessibility because you won’t find the same medicine from the same pharmacy then wakufana kuzoo, kana usina means dzekufambaka unokwanisa kuti lets say example kuti mumwe mushonga uri ku taakuenda…. Sezvataurwa na mhamha bigger challenge manje mari Because pharmacies are charging like, it’s like it’s in US dollars.

I: Uhmm.

R2: Ende if you convert it to RTGS dollars vari kuti it’s times 4. Whether it’s something chawaimbotenga ne dollar it’s now times 4.

I: Uhmm.

R2: Saka challenge yava kuti pa mari manje kana usina mari yacho it means kuti turnaround time yako kuti ukasike kupora it will take longer because pamwe uchamirira kuti mwedzi upere kana usina mwana anoshanda anokwanisa kukwereta mari it means wakutorwara more the greater part of the month usati wa healer.

I: Tiri kutaura nezvematambudziko amunosangana nawo kana muchitsvaka mushonga ye shuga.

All R: Uhmm.

I: Yenyu kana yevamunoriritira saka mhai vanga vataura kuti pano unogona kupihwa imwe kana vasina voti enda unotenga kune mamwe ma pharmacy. Asi kana wava kuenda kune mamwe ma pharmacy nhawu yemari ndooyanetsa kuti ma price emuma pharmacy haana kumira zvakanaka. Ndozvamuri kusanganawo nazvo here?

R3: Ehe mari mishonga yadhura.

I: Uhmm.

R3 Eee.

I: Saka inenge ichidhura iri muma bond kana kuti inenge ichidhura iri muma US?

R4: Inenge iri muma US ma US ndoorava kutoshanda. Bond racho ukanzwa ravanosheedzera unototya kana kuti uti...

I: Saka munoona zviri nani kutotenga ari ma US.

R4: Ma US acho kuti umawane price yacho iri kudeedzerwa mu market haasi kuita. Saka zviri kutorwadza mamwe ma test unototadza kuvaendesa test. Unoguma wadzoka sa gogo ivavava mamwe ma test handina kutovatoresa ini ndadzoka ndisina.

I: Uhmm.

R4: Ndichimirira kuti ndichivavarira kuti ndiwane chii matablets kuti vapote vachinwa apa ndiri kunyorerwa futi mamwe kutongozova kuti kumberi kwacho hakuna kumira mushe.

I: Uhmm.

R4: Unotochema ivo vanoti kudya chikafu chavo chavanenge vachida kudya chacho zviri kungorema.

I: Uhmm saka mukutenga muma pharmacy umu munomawana pa pharmacy yekutanga yamasvika kana kuti

R4: Ndinombotsvaga zvisiri zvekumbomira pa pharmacy one

R1: Uchimbocompeya ma price

R4: unomboti ukaenda apa wombonzwa price iyi wonzwa iyo, vachisiyana asi still hazvisi kubatsira nekuti vese vari kungodhura. Unozongoona kumwe kunge kuri nani asi mari yacho iri kurwadza. Panaapa pamwe aunenge wapihwa ndooemari shoma shoma iwayo e dollar dollar.

I: Uhmm

R4: Emari inodhura chaiwo Havana. Vanoti hatina.

I: Uhmm

R4: Idambudziko.

I: Ok mukapinda mu pharmacy mukashaya zvamuri kutsvaga, pharmacist arimo anokuudzai here kwakuwana anokuudzai here kuti tryayi apa?

R2: Ukasabvunza in fact ini ndinobvunza kuti munokwanisa kunge mume ruzivo here kuti ndinokwanisa kuwana kupi mushonga uyu vanotaura because vanenge vachizivana within the area kuti.

I: Uhmm.

R2: Kana kuri ku Avondale enda ku Avondale.

I: Uhmm.

R2: Ende unosvika uchiuwana mushonga wacho ikoko.

I: Uhmm.

R2: Whereas usakabvunzaka haakuudzi.

I: Ehe ndinokurudzirai kuti mubvunze nekuti ma pharmacist tiri muma watsapp group.

All R: Horaiti.

I: Saka munhu akashaya mushonga anogona unotogona kuona message nhasi pa phone pangu apa yakanzi une chakati mugroup rema pharmacist two hundred.

All R: Uhmm horaiti.

I: Anazvo anongoti ndinazvo 5 minutes.

All R: Horaiti.

I: Saka munogona kungobvunzawo kuti hamumawo here shamwari dzenyu dzamunogona kutaura nadzo pamaphone? Anobva akutsvagirai mukamubvunza eee anogona kutokuudzai kuti chiendai kwakati.

All R: Horati.

I: Saka ndanzwa muchitaura nyaya yema price zvakambobuda muma news paper munaana October umu ma pharmacy achinzi anyanya kucharger. Munofunga kuti ma price aya akakodzera? Nekuti vanotiwo kwavanozvitenga zvinovadhurira. Munofunga kuti zvakakodzera here kuti mishonga iite price yakadai?

R3: Uhmm zviri kunyanya mwanangu. Hatingati zvine kodzero nekuti vanhu vari kutenga vachoka vashoma vane mari vanoti I can afford.

I: Uhmm.

R3: Vazhinji kutamburira.

I: Uhmm.

R3: Sekuti nda admitwaka.

I: Ehe.

R3: Ndanga ndisinganwi mapiritsi for 5 days eshuga yacho, saka shuga ndobva yaita sei yakwira.

I: Uhmm.

R3: Ehe.

R2: Inin what I think is, in a way ka there are some chronic diseases zvekutoti like diabetes. Mushonga yacho haifaniri kudhuriswa because tiri kutarisa kuti munhu akaita two days asina amushonga wake.

I: Uhmm.

R2: Ava kuto affecteka. Ini a way vanofanirwa kutarisa kuti vanhu vacho vari kupihwa mishonga iyi.

I: Uhmm.

R2: Vakamira sei because ukada kutarisa vazhinji most of the people ma pensioners.

I: Uhmm.

R2: Havana mari.

I: Uhmm.

R2: Vanhu vatokura.

I: Uhmm.

R2: Saka vakakwanisa kudzikisa mishonga iyi kana kuti ye Diabetes kana kuti ye Stroke and the like.

I: Uhmm.

R2: Kana mamwe Ese ari very crucial then also kana… vokwanisa kunge ari accessible let’s say kunzvimbo yakaita se Parirenyatwa ineiyi.

I: Uhmm.

R2: Kana vachiti vari kuda ku curba corruption and the like vanenge vachitoziva kuti let’s say vanhu vachauya nhasi vangani then vo allocater ma pills anoenderana nema patients acho vovapa at a discounted rate because chikuru tiri kuda kuti vanhu vararame than…. we know we are in business but business racho harizofanira kuti woita markup ye at least 500%.

I: Uhmm.

R2: Uchikanganisa hupenyu hwevanhu.

I: Uhmm.

R2: Saka at least kana iri markup yavo yekuti variwo in business ngaivewo let’s say kana zvichinzi zvanyanyawo vachida kubhadhara rent kana zvichinzi zvanyanyawo 150% not 400% increase.

I: Ivo vanototi kwai vano markup yavo i50%.

R2: Aaa heee [laughs]

I: Hamufungi kuti ichokwadi?

R2: A good example handiti.

I: Uhmm.

R2: Uhmm ndoona kunge kunyeba a good example.

I: Uhmm.

R2: good example. Ini hangu handizoziva. There is this pill haa ok let’s take Panado.

I: Uhmm.

R2: Panado simple Panado kana ma MMT emudumbu aya aya.

I: Uhmm.

R2: Mamwe acho ano manufacture munoomu.

I: Ehe.

R2: Handiti.

I: Ehe.

R2: Achimanufacture munoomu you find kuti kana ari supplier anokwanisa kana kunomatora ku whatever industrial area I will not mention the name.

I: Uhmm.

R2: Ikoko unosvika unongoswipa pamwe at that dollar retail price.

I: Uhmm.

R2: Wosvika kunoku haachatarisa kuti no ndamatenga munoomu.

I: Uhmm.

R2: Ava kungocharger chii dollar US.

I: Ehe.

R2: Of which wakufanira kutenga ne 4 dollars bond saka zvimwe zvacho I think zvaa nekaelement kanenge kehutsinye so kekuti hatisisina hunhu huya hwekukoshesa varwere. Hameno pamwe hazvizirizvo.

R1: Ehe ichokwadi.

I: Ndiri kuona mhamha muri pa whatsapp.

R3: Ndiri kutaurira vana sorry.

I: Aiwa no musa apologiza nekuti mubvunzo wangu unotevera ndewe whatsapp.

R3: Hoo [laughs]

I: Ehe nekuti pane ma group ndaida kuti pane ma number amunokwanisa ku sender pa whatsapp mukati munotumira kuti ndiri kutsvaga chakati ndiri mu Harare.

R3: Horaiti.

I: Nditsvagireiwo pharmacy yezvakati woudzwa pa watsapp.

R3: Horaiti

I: Ehe, munoziva here kuti kune zvinhu zvakadero?

R3: Ndanga ndisngazvizivi ndanga ndisati ndambopapindawo.

I: Asi kana zviripo munogona utoshandisa here imimi? Munogona ku attacha picture ye prescription yenyu motora picture kudai?

R3: Ehe

I: Munogona kuto uploader ipapapa?

R3: Ndinogona.

I: Ndanga ndichida kubvunza futi kuti besides kudhura kana kunentsa kuwana kwauri kuita munomboita here kunetseka kuti mushonga yandiri kupuhwa ndeye chokwadi here ya expire here? zvinombopindawo mupfungwa kuti pamumotenga mushonga mu pharmacy mungangova muri kutenga mushonga waka expire? Kana kuti usina kunyatsogadzirwa zvakanaka?

R3: Haa tinongotenga

I: Munongotrusta kuti kana muchitenga mu pharmacy ndizvozvo?

R3: Ehe

I: Nemiwo munongo trusta kuti kana muchitenga mu pharmacy?

R1: Yaa tanga tisingazvitarisi zvechokwadi mamwe anenge a expire.

I: Makambosangana nazvo?

R1: Aiwa anga asiri evanhu vema bp ava anga ari mamwewo andaitsvaga.

I: Ndobva maona akato expire?

R1: Ehe ndobva ndatovaudza. Saka tavaudza… haaa kwanga kuri kuzvipatara kwedu kuma camp hospital ndobva ndabvunza kuti chii ma sisters acho ndobva vatoti kana pachine mwedzi kudai anogona kushanda.

I: Hoo ndoozvavakataura?

R3: Eee hanzi anogona kushanda so so wozoona manje

I: Saka makatoamwa?

R1:Ehe asi ndakazoita dambuziko ndamanwa.

I: Hoo.

R1: Ndoopandakaziva kuti kana chinhu cha expire hachifaniri kunwiwa ichi.

I: Saka makadzokera here kuti ndaita dambudziko ndanwa mapiritsi aya?

R1: Handina kuzodzokera.

I: Munoziva here kwamunokwanisa kuzvitaura? Kana mukatengeserwa mishonga yaka expire munoziva here kuti kune nzvimbo yamunogona kunozvitaura?

R1:: Hatizivi ndeipi?

I: Ehe iriko kungoti havanyayo zvishambadza zvavo but kuna Baines pa corner pa Third na Baines munongosvika ipapo mongoti ndine dambudziko nemushonga wandakasangana nawo ndauya kuzo reporter pa gate chaipo vanobva vatokuudzai office yekuenda. Panzvimbo pe white ne blue zi building ziguru re blue rakanyorwa kuti Medicines Control Authority muna Baines.

R1: Muna Baines?

I: Ehe kana mukaita dambudziko nemushonga mangopinda mu town munogona kudarika neko mongoti ndauya kuzotaura ndaita dambudziko nemushonga ndakautenga pakati dambudziko randaita ndeririri vanokubetserai. And munomboita here ma worries ema quality ema medicines amunotenga mu pharmacy?

R2: Eee usually ndinotarisa.

I: Munotarisa.

R2: Pese pandinotenga ndinotarisa.

I: Munotarisa chii? Exipry date?

R2: Yes because mostly anenge ari two three years saka ndinotarisa I will make sure.

I: Koo kana ari mu pill bag anenge akanyorwa papi?

R2: Anenge asina manje because pane pamwe pandakapedzisizra kutenga ku Avondale uko akangotorwa from that container muchicardbox.

I: Uhmm.

R2: Ndobva aiswa mu that plastic. Saka inini ndakangotrusta kuti since, ndanga ndatenderera for a long period of time.

I: Uhmm.

R2: Saka ini ndanga ndava kuda kukasika kuenda kumba ndaneta.

I: Uhmm.

R2: And I just trusted the pharmacist kuti zvaandipa izvi its ok.

I: Uhmm.

R2: But dai kuri kunzi achitipa asingaise muka plastic kaya kaya.

I: Uhmm.

R2: Achitipa the actual box because rino steta.

I: Ehe box rinotaura asi mune kodzero futi yekuti kana zvaiswa mu pill bag moti ndinoda kuona container yawazvitora.

R: Laughs.

I: Anobvumidza kungoti vamwe havazozvifaririi zvavo unoita seunonetsa

R2: (laughs) Exactly.

I: asi kana une ma suspicion zviri pamutemo chaizvo kungoti ndokumibirawo kuona gaba iro anobvumidzwa kukuratidza.

R2: Yaa we will try but zvinozoita kunge [overtalk]

I: Woita kunge uri kuva investigateR kana kudii ehe [overtalk]

R2: Kuita kunge usiri kutemba basa rake raakuitira.

I: Ehe hazvizobudi zvakanaka asi yaa ndizvozvo asi nyangwe mukaita problem nemushonga nyangwe mukaita reaction kana chii.

R2: Uhmm.

I: Ndanga ndichivatsvanangurira kunongoenda ku MCAZ monoreporter [overtalk] Its not getting someone into trouble pamwe pharmacist anenge asingazviziviwo kuti mishonga yake iri kukonzera reaction saka tinenge tichida kuziva kuti why is it causing a reaction saka zvakangonaka kuno reporter. Saka ndeapi ma solution amunoona kuti vakaita izvi zvingatibetsera, vakaita izvi zvingatibetsera?

R3: Saizvozvi handiti tinouya after 6 months tomboenda kuma clinic nekudii.

I: Uhmm.

R3: Vakatipa mvumo yekuti tipote tichiuya sesu tiri mu Harare.

I: Uhmm.

R3: Touya hedu kuzotenga mushonga unenge uripo pa pharmacy nekuti kuno tinoziva kuti wakati cheapeyi. Dzimwe nguva sevanhu tati kurei.

I: Uhmm.

R3: Topuhwa mahara.

I: Horaiti.

R3: Pane patakatombopuhwa mahara.

I: Uhmm.

R3: Asi pandakakanganwa chitupa ndakatenga asi kutenga kwekuno kuri nani.

I: Ok saka chii chinokukonesai kuuya?

R3: Tenge tisingazive kuti senge zvavanoita ku clinic kuti kana mune card.

I: Uhmm.

R3; Mune mvumo yekuuya munofanira… prescription paper iya iya… munofanira kuuya pa pharmacy motenga mushonga.

I: Ehe.

R3: Tenge tisingazvizive.

I: Heya. Ehe munokwanisa kuuya munongopiwa hospital number nyangwe musina kuonekwa pano, mopihwa hospital number pa casualty motoenda motonotenga. Ndatopedza hangu kutaura nemi asi kana pane zvemunoda kutaura zvinoenderana maringe nemushonga zvamunofunga kuti zvingangobetserawo.

R2: Ini I think kuti dai zvaikwanisikaka eee mushonga inowanika munoomu handisi kuziva kuti message ichasvika here to whoever is responsible mushonga inowanikawo munoomuka ngaingodzikawo kuitira kuti vanhu vakwanise kuiwana.

I: Uhmm.

R2: Yakadzika. Vasazoita zviya zvekutika vobva vakwidza zvekuti chero ukarwara nemudumbu unotozeza kunotenga mapiritsi emudumbu chero emusoro saka I think its...

I: Zvakasiyanei nekuti…. unogona kuudza munhu izvozvo oti vanhu veku public sector ndoo vanofanira kunge vachi dealer nekutengesera mushonga vanhu nemitengo yaka derera. Inini ndiri ku private sector and basa re private sector is profit ndoozvandakatovhurira ma door angu izvozvo.

R2: Yaa you are right.

I: Saka shuga yekwa OK kana iri kudhura endaiwo munovaudza kuti shuga yenyu iri kudhura dzikisai.

R2: We understand kuti vari ku private it’s after profit.

I: Uhmm.

R2: But let the profit ka not be something that can control you to the extend that you won’t see the human part to say these are humans also.

I: Uhmm.

R2: Because if you are ill give you a good example private doctor handiti.

I: Ehe.

R2: The private doctor akakoshesa mari kuti vanhu ngavandipe mari asingakoshesi the quality of work yaanenge achibatsira vanhu.

I: Uhmm.

R2: It won’t make sense saka anofanirwa kukoshesa quality of work and also the business side. Kuti zvandiri kupa ma patients angu aya.

I: Uhmm.

R2: Hazvibatsiri kuti unoita mishonga inodhura handiti.

I: Uhmm.

R2: Yotora lets say two years kuti izopera kutengwa. Pamwe pacho uchazopedzisira wava kutengesa mishonga yaka expire.

I: Uhmm, because haisi ku mover.

R2:Because haisiku mover. whereas vamwe vari kutengesa iri reasonable iri kutofamba. We know some other pharmacies mu Harare I think vanhu vakawanda can relate kuti pane ma pharmacies anotoziikanwa kuti aya akacheaper aya.

I: Uhmm.

R2: Wakasvika ipapo vanotononoka kuvhara vachiziva kuti taka cheaper and anofamba. If you go somewhere else.

I: Kwakanyarara?

R2: Kwakanyarara asi mushonga iriko asi ichidhura saka vanhu vakadaro manje ndoovanenge vava kutengesa mushonga waka expire. Later on vaakurealiser kuti no isusu we are now making a loss because hazvisi kufamba.

I: Uhmm uhmmm.

R2: So I think its something vakangoisa across the board ngavadhurisewo zvimwe like zvavanenge va importer somewhere else kwete zvemunoomu.

I: Uhmm.

R2: Yaa.

I: Ok. Gogo pane zvamunodawo kutaura kuvharisa musangano?

R5: [Clears throat] Chinonetsa ndichocho chekuti tiri kupihwa ma nhingirikini okunzi ma test.

I: Uhmm.

R5: Hanzi enda unotorwa ropa woenda wonotorwa ropa. Wochidzoka nema results acho. Tava muno tavakusvikopinda mune mume dokota asiri iye akandindinyorera kuti ndinotorwa ma test. Zvandava kunopinda mune mumwe uyo, haachandivhunza kuti koo ma tests.

I: Matests aya oh.

R5: Amakatorwa aya akazodai akazodai akadai. Hapana zvandichaudzwa nezvazvo.

I: Asi makashandisa mari kuitwa ma tests iwawo.

R5: Ehe

I: And moda kutozivawo.

R5: Ndoda kutozivawo. mari yakaburitswa ndopanmhidzwa futi mamwe ma test.

I: Aaa hii.

R5: Ndonzi endai futi kuma tests.

I: Ok.

R3: Ndoenda futi kuma tests ndekwekunokutura mari haumbobvisiswa tumari tushoma mari yakakura kwazvo. wochidzoka muno iye akakunyorera kuti enda kuma test hauchamuoni wakunopinda kune mumwe. Mumwe uya haakuvhunzi nezvazvo zvino kuzosvikaka ndaakuzoramba kutu aiwa ini zvema test kwete ndanyanya kutorwa ma test kana riri ropa aiwa ndanyanya kutorwa ropa. Unoona ini ndine goiter.

I: Uhmm

R5: Ndakarinzi enda unotorwa scan.

I: Uhmm.

R5: Ndakanokutura mari yakakura ndichifunga kuti scan yavo iyi ndikadzoka vanonditaurira kuti mukati me goiter umu manzi mune zvakati zvakati kana rinoda ku operatwa here, kana rinoda kupuhwa mushonga here hapana ndava kunosvika kupinda mune mumwe asirii ndiye haawoni nezve goiter zvo haachazvitaura. Zvino apa pakutorwa ma tests ini pachangu pava kundinetsa.

I: Hamuna here faira renyu rinogara pano rinonyorwa zvese izvo kuti nhasi maitwa chakati nhasi maitwa chakati zvonyorwa kuti anozongoriverenga anongoziva kuti last week makaitwa zvakati.

R3: Ndoozvandisiri kuziva kuti ndine faira here.

R1: Riripo book riya ravanenge vachiona kuti chiremba akambovaona vakadai ndoozvavanenge vachingoita. saana gogo vanogona kutadza kuita sei kuzvinzwisisa.

I: Horaiti.

R1: Chiremba anenge achitoona pazosiira mumwe wake.

I: Hoo horaiti haa its fine saka ndisati ndavhara zvakwana here zvamunoda kureva ndivharise zvangu.

R3: Aiwa vharisai zvenyu.

All: Laughing.

**Text C**

I: Sakaa sekutaura kwandamboita ndoda kungokurukura nemi pamusoro pematambudziko amunosangana nawo kana muchida kuwana mishonga ye shuga. Saka kuri kuti manyorerwa prescription pano chii chamunoita ? Munobva madii kana muchibva pano manyorerwa prescription?

R1: Takuenda kunotsvaga mushonga apo.

I: Papi pa pharmacy yepapapo?

R1: Ehe.

I: Ndoozvamunoita mese mese?

R2: Tinoenda kunotsvaga mushonga pa pharmacy ye panaapa asi dzimwe nguva imwe mushonga panenge pasina. Saka kana pasina pama pharmacy epanaapa tine challenge se last time kuti ukaenda kune mamwe ma pharmacy ekunze asiri epanaapa unonosvika kuchinzi kuri kudiwa ma US

R1: Kuri kunzi US dollar.

R2: Tainosvika tichinzi kunodiwa ma US mushonga hausi kutengwa ne bond.

I: Uhmm.

R2: Saka apa iwewe unenge usina US dollar.

I: Uhmm.

R2: Kutenga US dollar riya riya unenge une bond raitidhurira. Ndoo mamwe ma challenges ataisangana nawo.

I: nemiwo ndoozvamaisangana nazvo mukufamba?

R3: Eee ndasangana nazvoo tichiuya kunooku tichinzi hapana.

I: Uhmm.

R3: Asi mari yacho ndeye kutambudzikira muzukuru wangu haashande.

I: Uhmm.

R3: Ndeyekutambudzikira [coughs] zvaakutonyanya kudhura kuti ndichikwanise mariyo kwaakungodai kwakungodai vachitsvanzvidzira iwawa, wondipa ndoenda ikoko kuri kudhura maningi ndaa pano panenge pasina mapiritsi acho ese.

I: Baba ndoozvamunosangana nazvoo izvozvo, mukanyorerwa mushonga wenyu pano?

R4: Ehe mwana anosenza munoomu handitorina kutoti kupera kwemwedzi ndofonera ku Tanzania.

I: Hoo haasevenzi muno anosevenza ku Tanzania?

R4: Eee aaa.

I: Hoo saka ndiye wamunofonera? Obva atumira mari?

R5: Kuzoona mari yacho youya. Pamwe Haiuye ipapo ipapo inozouya pave payeka.

I: Ehe.

R5: Wozonotenga. iwe wanga wanzi tenga udye.

I: Ehoo.

R5: Handiti maona.

I: Ehoo.

R6: Plus imwe challenge yatinosangana nayo pamwe pacho vanenge vachinzi ngavadye ma fruits handiti.

I: Ehe.

R6: [overtalk] Apa chikafu chavanoda chacho tinenge tisina [overtalk] chikafu hupfu hwezviyo hwemhunga handiti vanonyanyoti ngavadye zve traditional zvakanyanya?.

I: Ehe.

R6: Saka ndoomamwe ma challenges atinosangana nawo kuti iwewe manje kumunhu ane shuga ukazomuudza kuti hapana mari manje nharo manje hamuzowirirane. [overtalk]

R7: Ukazotenga chimwe chinhu manje!

R5: Zvinonzi zvanga zvichinzi hapana mari asi mari yabuda nhasi.

I: Haa ok saka kana kuri kuti mashaya pano mushonga.

R5: Uhmm.

I: Mobva muchinotsvaga kune mamwe ma pharmacy.

R5: Uhmm.

I: Munoawana pa phramacy yekutanga yamunosvika?

R5: Haa dzimwe nguva unoawana ukaita lucky zvine mazuva.

I: Horaiti.

R5: Dzimwe nguva unsvika wonzi hapana woenda kana patatu uchinzi hapana.

R6: Inini ndinoona muma pharmacy challenge zhinji yatinoona muna pharmacy ndeye ma piritsi e bp, eshuga kazhinji kacho haanyanyo netsa but e bp manje.

I: Uhmm.

R6: Nemamwe acho eshuga anofambira handiti anofamba dzimwe nguva unenge wanyorerwa ari 5.

I: Ehe.

R6: Unogona kuwana ma type three kana kuwana two.

I: Uhmm.

R6: Wobva waenda kune imwe pharmacy price manje ndoo inoita kuti usiye nyangwe wanga wa arimo [overtalk] nyangwa dai wanga wamawana nekuti mari yacho inenge isingakwani. [overtalk] Saka price yacho manje unobva wafunga kuti musi wanenge avamo mu pharmacy ndongozodzokera ndoita sei ndonotenga of which ava havachanwi mapiritsi.

I: Hoo vava kungozvibaya?

R6: aiwa day iroro vanotojumba nekuti mari yacho inenge isingakwani.

I: Heeya.

R6: Apa unenge usina option nekuti pharmacy iyoyo inogona kuti mapiritsi edu tinomawana manheru kana mangwana makuseni.

I: [Overtalk] Vanorega?

R6: Day iroro vanotojumba nekuti mari yacho inenge isingakwani [overtalk] apa unenge usina option nekuti pharmacy iyoyo inogona kuti mapiritsi edu tinoawana manheru kana kuti mangwana kuseni.

I: Uhmm.

R6: Saka iwewe unozongomukirawo uchingotenga ikoko pamwe panenge patorine musiyano we 2 dollars kana kuti we 3 dollars.

I: Ehe haa horaiti saka kana kuri kuti manzi hatina masvika mu pharmacy manzi hatina vanokuudzai here kwaunogona kuawana kumwe kana kuti vanongoti hatina?

R5: Pamwe vanotaura endai pakati.

I: Ehe.

R5: Woendawo pakati pacho wosvikoshaya futi.

R6: Pamwe pacho vanogona kukudzai kuti endai ku pharmacy yakati handiti.

I: Uhmm.

R6: Masvika imomo sekutaura kwandamboita kuti ma challenges atinozosangana nawo ndeema price manje

I: Ehoo

R6: Masvika imomo unogona kumawana achinzi arimo mapiritsi but price yawo ndiyo inoku controlla wega kuti umbomirira uku anonzi ari kuuya manheru kana mangwana makuseni.

I: Saka zvingakubetserai here kuwana nzira yeku compeya ma prize wakagara mumba mako uchicompeya?

R6: Ini ndinoona kunge zvinotobatsira zvekuti dai taiwana kana kuti zviya zviya zveku senderwa pama phone kuti unogona kuwana ruzivo rwekuti ndikashaya mapiritsi angu kwakati ndinoenda kwakati ma pharmacy anowanika mapiritsi nguva zhinji. Nyangwe dai ane ma price ari pamusoro.

I: Uhmm.

R6: Asi uchikwanisa kutenga mapiritsi unogara waka budgeta kuti kupera kwemwedzi ndinonotenga mapiritsi emari yakati pa pharmacy yakati.

I: Unenge watoudzwa pa phone [overtalk]

R6: Asi ese ndinoawana.

I: Uhmm.

R: Nguva imwechete.

I: Hoo.

R6: Pane kuti watenga mu pharmacy iyi wopinda mune iyi wopinda mune iyi.

I: Uhmm.

R6: Zvinoticostera mari yakawanda.

I: Makambofungawo here kuti mushonga yamunopihwa mu pharmacy haina kukodzera pamwe yaka expire kana yakagadzirwa nemhando yee kana munongoti kana zviri mu pharmacy zvakachena?

R6: Inini hangu kutaura chokwadi ndaingoti kana zviri mu pharmacy sezvo une ma pharmacist pamwe zvinenge zvakanaka zvekutarisa ku expire haa kashoma.

I: Kashoma.

R6: Unonogotarisawo pabepa paya paya panenge pakanyorwawo tisingazive kuti anenge aka pekwa acho anenge agara aka expire here?

I: Uhmm.

R6: Kana kuti anenge ari up to date here hatizivi.

I: Uhmm.

R6: But chatinoziva ndechekuti pharmacist ndiye atipa chii mushonga saka taakutonomwa

I: Haa zvakanakayi nekuti takambozwa mwedzi yapfuura iyi ma pharmacist achiitukwa chaizvo pa TV nepa Radio nyangwe President vakataura kuti vanhu vari kutengesa mushonga nemitengo isina kunaka.

R4: Ne USA.

R6: Yaiva nyaya yema USA.

I: Ehooo ehe, saka makaona zvakashanda here?

R4 :Hazvina kumboshanda.

R6: Asi kutaura kwakaita President vachiti mishonga ngaitengeswe ne bond inini ndinoona kunge zvakatibatsira because ikezvineezvi mishonga mizhinji takuitenga nema bond. Kozotiwo mimwe vanhu vemuma pharmacy vanototaurawo kuti mishonga iyi isusu hatisikuiwana muno muZimbabwe. Saka kuti tikutengeserei nema bond isusu tiri kuiwana kunze.

I: Uhmm.

R6: Hazviite saka tinenge tichidawo ma USA ivo vanogona kungo converta bond rako iroro voriisa USA. [overtalk]

I: Hoo saka moda kuti vatore ma bond enyu vaconverte voga kuita ma USA [overtalk]

R6: Ehe vanotodaro kuti pa dollar rako tava kukuti 4 dollars kuitira kuti isusu tigonotenga mushonga uyu nekuti hausi wemuno.

I: Haa ok ndanzwisisa.

R6: But inini hangu ndinoona hangu kunge zvinotibatsira pakuti mishonga yacho iyoyo inenge ichinzi haisi ye munoomu of which ukauwana uka convertwa kuti iwoyo nema bond iwowo ndoowoiswakuma USA zviri nani pane kuti ndinotsvaga USA nekuti handizivi kwandinoriwana.

I: Hoo saka imimi munoti vanoziva kwavanoriwana asi imi hamuzivi kwazvinenge zvichibva?

R6: Tinenge tisingazivi kwazvinenge zvichibva manje.

I: Horaiti.

R6: Dzvimwe nguva unoti ubude utsvage USA wakuto vhunza iye mupurisa wava kutosungwa.

I: Heeee.

R6: Kuti ndiri kutsvaga USA saka ndoriwana papi [overtalk]

I: Saka mune ma phone anoita watsapp here?

R (several): Ehe tinawo.

I: Nekuti usually pama watsapp ndoo paunoona information iyoyo yekuti kwakati zviri kuita marii mushonga urikuita marii kwakati unogono kutouwana ende mu pharmacy ukapinda akati hatina unogona kukumbira iye pharmacist kuti imimi mune mabhururu enyu epama whatsapp.

R: Uhmm.

I: Hamugoni here kubvunza ende vanozviita, izvozvi ndinogona kutovhura phone ndoona ma message anenge ari pa group pharmacist atoisa message kuti hapanawo here ane chakati pane ari kuchitsvaga mu pharmacy mangu ongomira 5 minutes achipihwa response then wozoudzwa kuti chiendai pakati vati vanazvo wobva watobatsirikana saka maphone anogona kushanda zvakati ooo iwo mawhatsapp iwaya kana muchigona kutumira kana picture prescription yenyu motumira pa watsapp ipapo motoudzwa kunzi aaa mushonga wenyu unowanika kwakati unoita marii asi musina kuu. Zvinhu zvamungatoda here nekuti munogona kutozvibhadharira dhora izvozvo?

R: Zvekuu?

I: Zvekuudzwa kuti mushonga uri kwakati unoita marii?

R6: [overtalk] Izvozvoka zviri right nekuti ndinenge ndichikwanisa kuwana mushonga wacho wese kaone panguwa imwechetee. Hazviku costiri nekuti uchafamba uchati nhasi wapinda apa mangwana wapinda apa mangwana wapinda apa.

I: Uhmm.

R: Apa ndikawana mushonga wacho kaone nguva imwe chete zvinenge zvirinyore.

R: Zviri nani.

I: Pazvakanzi ma pharmacy ari kudhura maningi ivo vaiti public sector kunaana Parirenyatwa zvipatara zve hurumende.

R: Uhmm.

I: Ndookwamunofanira kuwana mishonga yakacheaper.

R: Uhmm.

I:Kuno mukauya kuno tinotamba bhora reku private.

R: Ehe.

I: Ndoozvatakatovhurira ma shop iwayo kuti tiwane kurarama.

R: Ehe.

I: Nekuti mataurire amunongoita ekuti kana maenda kwa OK kana shuga ichidhura hamumbofi makaudza President kuti enda kunoudza OK kuti shuga yake yadhura.

R: Uhmm.

I: Saka sei muri kuti zvedu zvidzike zvakasiyanei?

R: Imimi kuda kuti batsira isu tiri varwere ka isu.

I: Ehe.

R: Tiri varwere kuti batsiraka.

I: Kukubatsirai?

R: Ehe dhurai zviri nani haikona kubva madhura zvinobva zvarwadza.

I: Uhmm.

R: Ehe.

I: Ok.

R: Ehe.

I:Saka muri kuti musiyano we mushonga neshuga ndeyekuti mushonga unoraramisa shuga haina mhaka shuga yema OK haina mhaka ikashaikwa haa.

R: Haa sister mushonga wakakosha.

R: Ehe.

I: Ndookusiyana kwazvaita kuti chacho chiri kutengeswa mushop [overtalk]

R: Mushonga hupenyu ka shuga ndikaishaya haurware nekuti hauna kumwa tea.

R: And shuga ndogona kuisiya ndonobika sadza but mushonga handikwanisi kuusiya nekuti ndinoda kurarama.

I: Ok Hoo ndanzwisisa pane nyaya yacho aa tatopedza unless kana mune zvimwe henyu zvamuri kuda kungo edha zvamunoda kutaura zviri pakati pemoyo wenyu kuti dai zvaitwa izvi zvaitibatsira izvi dai zvaitwa.

R: Dai pano chete pachinoita mushonga yatinowana tinorarama tinopona.

R: Tinotambura.

R: Ehe mushonga inotinetsa muma pharmacy umu mune basa dhora pa 4 dollars ipapo ukaita 4 dollars fanika bp unopuhwa ka one katsete kapacket so karefu so one ke 4 dollars.

I: Uhmm.

R: Kuti upihwe inokwana mwedzi vanotoda chii 16 dollars kuti ikwane mwedzi.

I: Saka mukapinda mu pharmacy kana kuti mukauya kwachiremba munopihwa here imwe information inenderana nemapiritsi enyu munoudzwa here kuti ah mapiritsi atakupai aya anokonzera chakati munoona mava kunyanya kukosora anokonzera side effect yakati mukaona zvadai mudzoke. Munopuhwa here humboo ihwohwo, munoziva here kuti mushonga wamunomwa unoita nezvei unonzi kudii uye ma side effects aunogona kukonzera ndeapi munotsangurirwa here zvese izvi?

R: Uhmm umm umm hatisati tatsanagurirwa.

I: Mungada here kutsanangurirwa kana kuti hamunwo basa nazvo munenge muri kuda kunzwa kuti munotora kangani.

R: Aiwa ndinoda kuziva kuti ndiri kurapwaka ndiri kupora.

I: Uhmm.

R: Zvekutu mushonga uyu unokonzera zvakati ndikazviziva ndichanwa mushonga wacho?

I: Saka hamutodi kuzviziva?

R: Aaa ndikazviziva ndichada kunwa?

R: Zvakafanana nezvakaita nhasi.

I: Ehe.

R: Nhasi chiremba wandasangana naye ku shuga.

I: Ehe

R: Wanyatsoti explainera zvekusvika pakunyora prescription. Apezda kunyora prescription abva atanga kutiudza uti uyu mushonga uyu.

I: Uhmm.

R: Ndewe shuga, unoita kuti shuga idai idai idai uyu ndewe bp.

I: Uhmm.

R: Unoita kuti bp idai so.

R: Ehe.

R: Uyu ndanyorera kuti musazo stroker.

I: Uhmm.

R: Saka mishonga yese iyi hamufanhiri kuishaya saka ndorumwe ruzivo runenge ruchibva kunaana chiremba zvekuti tinotenda nekutibatsira kwavanoita.

I: Mungatoda kuramba muchiudzwa information iyoyo.

R: Ehe.

I: Asi yema side effects hamudi kuihwa?

R: Uhmm ini handidi.

R: Yaa iyo yekuti mushonga unozokuvadza zvakadini muviri.

I: Hamutodi.

R: Information iyoyo yava yakoo na chiremba. Yaakurwadza iyo.

I: Ok ndanzwisisa argument yenyu.

R: Ndoda kutsvagirwawo phone number yemwana wangu ndoda kumuudza kuti ndapedzerwa ari kunditsvaga uyu.

I: Ehe endai henyu munotsvaga.

**Text D**

I: Aha.

R1: Saka mishonga yacho mapiritsi acho iri kudhura you see kuti quotation yavo ndeye 70 US.

I: Pano munenge musati waiwona here mishonga?

R1: Panaapa pane inowanika pane imwe yekuti hauiwani but panaapa pacho sometimes pano panoda kubhadharwa but handizozive hangu kuti quotation yepanaapa inenge iri marii like nhasi ndatouya handina kana mari kuzongoenda apo pamwe ndongonyorerwa ndobuda.

I: Uhmm.

R1: Ehe.

I: Koo imi zvakakurerukiraiwo sei?

R2: Inini ndinochengeta murwere we shuga issue ndeye mapiritsi iyoyi but last time patakauya takaaona zvakanaka.

I: Ehe.

R2: Hatisi kuziva pari nhasi kuti zvakamira sei [overtalk]

R1: Makabhadhariswa here unenge uchingotengeswa?

R2: Ehe takatenga.

R1: Koo zviya zvamaimboita Dudzai zvekuti old age you see mai vangu chaivo ndeva 37 zvekunge vachipihwawo imwe hazvichatioiti takufanirwa kutongobhadhara?

I: Maover 65?

R1: Ehe.

I: Havapuhwe mahara here?

R1: Ndinongobhadhara ini.

I: Munongobhadhara?

R1: Ehe.

I: Asi nofunga kuonekwa ndookwamuno wonekwa mahara asi mushonga munobhadhara handisi shuwa zvangu ndichabvunza.

R1: Ehe.

I: But I think..

R2: Mushonga unobhadharwa handiti?

I: Ehe. Aaa ok saka kana kuri kuti mari munayo inowanika kuma pharmacy ese amunobvunza here?

R2: Ehe inowanika asi kuzoti kuma pharmacy manje iri kudhura inenge yazosiyana mari pane.

I: Pane yepapapa?

R2: Ita kuti sema paharmacist itai kuti mishonga dzakawanda dziwanikewo pano nekuti kana munhu anyorerwa mushonga tinotengawo nemari iri nani pano.

R1: Pane imwe inodhura inonzi Giblimide panaapa haitombowanika.

I: Uhmm.

R1: Haitowanika zvachose pano unongonoiwana kupharmacy uko ndoo inenge iri 28 US iri one kapacket kana kuti 30 US iri one.

R2: Asi matauriro atiri kuita makutozivaka kuti mishonga yakati dai ichiwanika pano.

I: Uhmm.

R2: At least dzimwe mari dzinenge dzakaderera.

R1: Uyu (shows moderator a medicine packet). Kanodhura kamushonga ikaka zvisingaite.

I: Ehe glimepride.

R1: Ehe nekaka.

I: Iyo ichii iii?

R1: Ndofunga I 8 US iyoyi.

I: Hoo.

R1: Saka panaapa hauiwani. ungatowana ana Enalapril naana Naladapil chii chii HCT ne Metformin.

I: Uhmm.

R1: Iyi inodhura yachoo iyi unenge wakaomerwawo worse,

I: Hooo.

R: Zvekuti kana mwedzi waakutopera unenge utori papfungwa kutaura chokwadi ndipedzisira ini ndatove ne BP woo.

I: Uhmm.

R: Ehe.

I: Koo imi baba?

R3: over 65 years vanowanza kupa mishonga.

I: Munowanzopihwa mahara?

R: Ehe.

I: Munovanzwaka kuti ma over 65 vanowanza kupihwa mahara.

R: Kupi?

R3: Pa pharmacy panaapa.

R2: Pharmacy ipi?

R3: Haa handichaziva hangu kuti ndeipi.

I: Pane pharmacy ka ipapapa.

R: Ehe ndakapihwa kaviri ndine 66 years hapana chandakabhadhara.

R1: Makatanga kubva kudhara?

R: Ehe.

R1: Kubva kare kare?

R3: Last month ndakapuhwa last week ndikapihwa.

R1: Aiwa handisi kutaura zvemanje manje pese apa pamwe vaakupa ini handizivi because taaene 6 months tisingapihwe.

R3: Haa varikupa.

R1: Imi munoziva sei muriimi.

I: Ini handishandi pano. Ndinonziwo ndiwongorore zvandinowana ndovaudza.

R1: Maover age ka dai vachingopawo.

I: Ehe.

R1: Nekuti tinozova kuti inotengwa dai vachingopawo imwe yavainayo.

I: Ehe.

R1: Kuitira kuti patinobudawo kunze. Vanofanira kuita makore avanoti aya akatiwo.

I: Hoo.

R1: Ehe.

I: Saka muripa medical aid here?

R3: Inini?

I: Mose?

R1: Aaaa ini hapana medical aid.

I: Hamusi saka motoshandisa mari yenyu kuu [overtalk]

R1: Havana medical aid amai vangu.

I: Hoo ok saka kuri kuti mawana kwakacheaper ndookwanunotenga nyangwe customer service yavo isisna kumira zvakanaka?

R (several): Uhmm.

I: Hamuna basa ne customer service?

R (several): [laughs]

R: Ibasa.

I: Saka mukanzi ava vakacheaper asi vane bad customer service ava ava vanodhura asi vanokubatai zvakanaka munopihwa pekugara [overtalk]

R2: havazomboite nhigirikini because ava vanenge vachidawo mari ikoko kwacho.

I: Havaite bad customer care?

R2: Havaite rough because vanotoita vachitoziva kuti ma customer anoto tiza saka vanotobata vanhu zvakanaka.

I: Uhmm saka motongoenda kwaka cheaper.

R2: Saka totongoenda kwaka cheaper, asi akawanika panaapa zviri nani because tinongoziva kuti tikabva ma chiremba.

I: Uhmm.

R2: Takunotenga mushonga dzedu tobva tadini tobva taenda.

I: Saka imo muma pharmacy munomboitawo here dambudziko rekufunga kuti zvandiri kupuhwa izvi pamwe ndezve fake pamwe zvaka expire kana kuti munogoti pamauwana mauwana?

R: Haa ipapa hatisati tasangana hedu nedambudziko iroro. [side-talk unrelated to research]. Mapharmacy anenge ava ekutongoonawo…unofanirawo kuendawo mupharmacy ekuti aya handifungi kuti anombotengesa zvinhu zvakaexpire.

R: Zvasiyana nemusupermarket ka.

I: Munoziva sei kuti iyi pharmacy haingambotengesi zvaka expire munenge mawonei?

R: Unozongotarisa handiti unopihwa mushonga wotarisa ma date.

I: Hoo.

R: Kana ne problem wototi koo hindava ma date akamira sei?

I: Uhmmm munoziva here nezve Diabetes Association yemu Zimbabwe. mubatanidzwa we vanosimbirwa ne Diabetes makambonzwawo nezvayo?

R: Takambonzwawo semataurire auri kuita asi handina kumboiunderstanda hangu kunzwa kuti zvakamira sei.

I: Uhmm.

R: Asi ndakanzwa kuti iri inojoinwa unozokwanisa kumiririrwa kuti uwane mushonga inojoinwa ne 50 dorrazi ndakangozvinzwa but handina kuzombozvitedzera.

I: Hamunakuzombozviteera kuti zvinoitwa aaa ok.

R: Ehe.

I: Haa sorry tanga tichitaura nemishonga yevanhu vanosombirwa ne Diabetes kuti zviri nyore sei kuiwana zvakaku cheapirai sei ndozvatanga tichikurukura handizivi kuti pane zvamunoda kutaurawo here pamusoro pema experiences enyu?

R: Haa no handina.

I: Haa its alright makadii baba?

**Text E**

I: Taurai henyu.

R1: Mishonga yacho tiri kuishaya.

I: Uhmm.

R1: Yakakwira dzimwe nguva tiri kutadza kuitenga iri kunzi iri kuda ma USA saka zvimwe zvachona zvaakuti netsa ndezvekuti mishonga mizhinji hatina.

I: Uhmm.

R1: Yeshuga panaapa unogona kuwana mamwe ma tablets esghuga unogona kuishaya. Unogona kunwa one tablet instead yekuti unwe two unenge usina mushonga. Matablets izvezviizvi tava kutombotrumira dzimwe hama dzirikunze uko votitumira but mari yacho tinenge tisina. Ukawanawo ukashaya hatina tikawana toshaya tikawana toshaya mushonga hakuna.

I: Uhmm saka iri kutoshaikwa nyangwe une mari yacho?

R1: Mari unenge uinayoka asi inogona kuita shoma pane iri kudiwa. ma pharmacy mazhinji anotengesa nema USA and mushonga umwe chete kuti u afforde uri kuda mari yakawanda apa unenge une two types dzema piritsi.

I: Uhmm.

R1: Wotaurirwa mari yakawanda pamwe mari yacho unenge usina saka mushonga hatina yeshuga.

I: Uhmm.

R1: Tiri kuishaya hatisi kuiwana. Kana tikanyorerwa mushonga kunooku mamwe ma tests aunege wanzi unoita mamwe acho haaisi kuitwa nekuti vacho vari kuti hatikwanise kuita tsvagai mamwe machiremba anokuitirai. Machiremba anenge achidhura zvekuti unopedzisira usina imwe test yawaitwa.

I: Uhmm saka kurikuti tomboti musi wamunenge mune mari munouwana mu pharmacy yamunotanga kupinda here kana kuti munofanira kutombotanga mafamba muchitsvaga?

R1: Unotombotanga wafamba uchi compeya kuti muri nani ndemupi. Ukasvika munenge muri nani ukambo compeya compeya ukaona muri nani ndoomaunodzokera kunodii kunotenga.

I: saka munotsvaga kwakacheaper nyangwe customer service yavo isina kumira zvakanaka? Tomboti kwamusingabatwe zvakanaka asi wakacheaper ndookwamunotongoenda?

R1: Hapana zvekuita tinononotenga nekuti tinenge tisina choice. Hatina choice.

R2: Ndizvozvo unenge wakutenga kucheaper mwanangu, kucheaper hazvinei kuti ndabatwa sei handiti.

R3: Damdudziko guru nderekuti ndaona zvaka chaper regai ndinotenga chero ndichitukwa chero ndichidii hanzvinei handiti. Then imwe point yataura futi mhamha yema nhingirikini yema tests like H chii?

R1: Haisi kuitwa iyo.

R3: Hanzi H chii handichazive.

R:1 Yakanzi HBS?

I: Hoo HB1AC?

R3: Ehe munoziva ndakaiitwa day before yesterday?

I: Uhmm.

R3: Ne 48 dollars.

I: US?

R3: Aaa ndakanga ndanzi 15 bond sorry 15 US then pama bond ndoopandakanzi 48. 48 dollars yachona ndaiwana kupi handiti. Ndinoda kutengera vana chikafu.

I: Uhmm.

R3: Vana vari kuda kuenda kuchikoro saka tinorarama sei kana kupenyu huchiita so mwanangu?

R4: Haa mamwe ma tests acho hatichaiti isu nekuti tinenge tisina mari yacho.

R3: Ndakatombofunga kuti rega ndiregedze handiti? Ndatoona kuti ka mari aka kakabuda vana havasisisina chekudya ndobva ndazodzoka futi ndochifunga kuti utano hwangu hupenyu hwangu.

I: Uhmm.

R3: Ndoosaka ndakazoitwa.

R5: Hupenyu hwacho huri kuoma.

I: mmmm

R5: Ipo pahupenyu hwedu hweshuga apa.

I: Uhmm.

R5: Hupenyu hwedu hweshuga takatsika parufu handiti.

I: Uhmm.

R5: Nekuti tinofa anytime. Upenyu hwemunhu ane AIDS hwakatosiyana nehwedu.

I: Uhmm.

R5: Taiderowoo muchititsvakira donor unotipawo ma injection namapiritsi eshuga. Ini ndinozvibaya shuga ndinotenga injection ndotenga mushonga. Pamwe pandinenge ndawana mari ndichida kutenga zvibhotoro zviviri zvemushonga zvinonzi ndinongokupai one, asi ndinokwira bhazi ndonzi 20 dollars kuenda nokudzoka.

I: Munogarepi mhamha?

R5: Kumusha kwa Gutu saka zvokwadi kutongomirira kufa mwanangu.

I: Saka munobva kwa Gutu kuuya kuno ku Parirenyatwa?

R5: Kuno ku Parirenyatwa ndookwayakabatigwa kana ndikati ndoenda ikoko kwa Gutu kungotarisa chete kudai vanoti 20 dollars wozotenga mushonga wamboonekwa ne 20.

I: Kuno havabhadharisi kuonekwa?

R5: Kuno ndinobhadhara 10.

I: Ehooo.

R5: Eee hazvirwadzi here? ndinoiwanepi? Wozotsvaka imwe mushonga wadhurirwa kudero. ende zve kwa doctor anotanga ku…. wainda iko kuya vondokubata rough munombhodaro sei. Saka zvinonetsa. Zvinonetsa. Shuga yedu yanga ichida kutsvagigwa donor mushonga yedu kuti tiraramewo.

I: Uhmm.

R5: Kuti tiraramewo.

I: Saka kana muri muno mu Harare munogarepi?

R5: Kuno ku Harare ndabva ku Hatcliff kumuzukuru uriko. ndokwandakabva zuro riya Monday manheru ndikavata ikoko.

R6: Ndisingazive kuti pharmacy yepapanaapa hanti ndiwe uri panaapa mwananangu?

I: Aaa ini ndakadanwa kuti ndingoita ongororo ndichazovazivisa zvamungenge mataura.

R6: Aaa ndanga ndichida kubvunza kuti pharmacy yepanapa Insulin vanayo here? Une idea here?

I: Aaa zvanhasi handina kuvhunza vanosiiita here?

R6: Handizivi mazuva ese ndinotenga muma pharmacy mu town handisati ndambotenga pano.

I: Hamusati mambotryer apo?

R6: Handisati ndambotryer apa.

I: Saka munoti mukanyorerwa motongoenda hamubvunze?

R6: Ndobva ndangoenda handisati ndambovhunza ini.

I: Koo munoregererei kungopfuura muchivhunza?

R7: Vanoti hapana panaapa.

R6: Ndakambovhunza kamwe chete zvikanzi hapana ndobva ndangoti haa ngoma ndiyo ndiyo, saka ndakabva ndatosiyana nazvo ndobva ndati zvinongova zvimwe chete izvo. Chero ndikabvunza kungopedza nguva.

I: Taurai henyu ndaona makasimudza ruoko.

R8: Zviri kutinetsa ini hangu ndine shuga but it’s not that high.

I: Ehe.

R8: Asi adzimai vangu it’s high, tiri kusangana nema problems kuma pharmacists kwenyu ikoko, vari kuda ma USA asi isusu hatihore ma USA hanti mazviona.

I: Uhmm.

R8: Although medical aid vava kuiramba futi ini ndine private Cimas but vari kuiramba futi saka ndoo dambudziko iroro.

I: Ivo vanoti kwavanotenga mishonga iyi vanobhadhariswa ma USA vanotenga kunze kwenyika havanzwisisi kuti vozdiiwo because vakakutengeserai vanoda kuwana kureplacer zvamatenga kuti vazotengeserawo vamwe kana imi mwedzi unouya saka vatoriwo panguva yekuti isusu takawomerwawo nekuti kwatinotenga tinotenga..

R8: Ndoo pane dambudziko ipapo. Ndosaka pamati tikubvunzei ndafunga kuti pamwe….

R (unidentifiable): Dai yawanika panaapa mishonga ikangowanika panaapa tinotenga zvakanaka [overtalk]

R9: Isusu tinenge tava kutoti tava kutochemawo ne hospital kuti kuma pharmacy isusu tiri kuwomerwa nekuti US racho hatina kwekuriwana saka hospital kana ikange iine mushonga panaapa isu tinongouya nevarwere vedu tichitenga mushonga pano nekuti zvinenge zvakatirerukira nekuti hospital tinenge tava kuziva kuti ihospital yehurumende tinogosvikotengawo ne bond riri kudii riri kushanda panekuti ndiende ku pharmacy ndononzi tiri kuda US. US racho ndirikufanira kuritenga. 10 US kuti ndiritenge ndiri kuritenga ne 35 saka muri kungoona kuti nemararamire atirikuita zvakadii zvakaoma tinopedzisira takuuraya vabereki vedu vanenge vachifanira kuti vararame nenyaya yekuti hatisi kukundikana asi kuti tiri kuwomerwa nekuti zvinhu zvacho zvanyanya kuwoma.

I: Saka kuri kuti maenda kuma private pharmacy munoawana pa pharmacy yekutanga here kana kuti munotofanirwa kufamba muchi bvunza?

R8: [Overtalk] fanika last week ndakaswera ndichifamba the whole day hapana pharmacy yandisina kumboenda kusvika ndazoenda ku ***** ndobva ndazonzi ndinofanira kuenda ku *****.

I: Uhmm.

R8: Ku Borrowdale ndookwandakazouwana but ikoko zvavo havana kuita sei havana kutaura US vakangotaura bond asi bond racho [laughs]

R (unidentifiable): Bond racho kunenge kuuraya munhu

R8: vaingoda ka jekiseni kakadai vanga vari muchipatara, ka jekiseni kakadai so 122 dollars saka zvinhu zvekuti kana usingakwanise kuti murwere…. zvinonetsa unoiwana kupi mari iyoyo kana kurikuti vamwe vasingaende kubasa.

I: Ehoo saka munenge mava kutongotsvaga kwakacheaper?

R8: Hapana kwakacheaper.

R9: Hapana kwakacheaper mazuvano murikuita kunge muri kuudzanaka.

R: Ehee.

R9: Nekuti uyu akakwidza I two minutes mumwe anenge atokwidzawo unoshaya kuziva kuti zvakwira nguwai. isusu tiri kutochemawo nemi mapharmacy acho kuti hamumawo here zvamunogona kuita kuti imimi muudze hurumende kuti zvinhu zvakatiwomera kuti vawone zvekuita aipapaapa pagadziriswe.

I: Zvinenge zvichinetsa nekuti vari kuda mari yemushonga vari ku competer nevarikudawo fuel ku Reserve Bank pane vari kuda fuel nevari kuda mushonga.

R9: But mushonga uyu mushonga une hupenyu hwevanhu ka uyu fuel ndeye kufamba zvedu zvimwe zvacho ndezve luxury but hupenyu hwevanhu uhu. eee.

R: Ndoopane dambudziko.

R10: Ini hangu ndinofunga kuti dai hurumende yangoita senge zvainoita pama piritsi e HIV.

I: Hoo.

R10: Vopota vachipa vane shuga pachena tongoziva kuti tichanotora mushonga wedu.

R9: Especially iye zvinezvi vanenge vachiti old age but old age yacho tiri kungonotenga mapiritsi ma piritsi acho tinenge tapedza pasi kuti tiawane saka ngavaite zviri nani zvinogona ku affordwa nemunhu wese.

I: Uhmm.

R9: Kune vamwe vabereki vedu vekuti havatorina mwana wacho anogona kuvatengera mapiritsi.

I: Uhmm.

R9: Ndoovega vari kuti kavanenge vakasungirira kaya kaya ndookauri kuda kunotengesa munosvikonzi pari kudiwa US vanoriwana kupi?

I: Uhmm mareva mushonga we HIV unopiwa mahara vanogona kuti yeHIV vanopuwa mahara nekuti pane mutero uya unonzi AIDS levy ka.

R10: Vangadai vakaitawo izvozvo.

I: Saka tinenge taane mumwe mutero?

All R: Ehe.

I: Mungauda here mutero imi?

All R: Chaizvo.

R10:diabetes inouraya iyi saka ngakungoitwa mutero we shuga.

I: Mutero we 2% wakatitsamwisa sei wani moda kuti uwedzerwe futi?

R10: TB inorapwa ichiri kurapwa mahara here kana kuti?

I: Inorapwa mahara ehe.

R (several): [overtalk] saka shuga todii kurapwa mahara

R10: handiti muri kuona saka tiri kungo categorisa zvinhu, zvinhu zvakafanana zviri deadly [overtalk] ngazvingoitwa tirapwe zvedu mahara because hakusi kuda kwanguwo.

I: Ndazvinzwa zvamataura zvese zvinogova zvimwechete. Taurai henyu mhamha.

R11: Ndoda kutauraewo mwanangu.

I: Ehe.

R11: Kuti isu mapiritsi ari kushaika mabond tiinawo kuma pharmacy kwacho tiri kushaya mapiritsi hatisi kumawana mapiritsi ari kungoshaika.

I: Uhmm.

R11: Eh. Eshuga ne BP hatisi kuawana chichemo changuwo ichocho hameno vamwe kuti vanonditsigirawo here?

R10: Unozoawana watenderera taundi yese.

I: Saka kuri kuti kwagadzirwa chinhu chinoshanda pa phone chekuti chinokuratidza kuti mushonga waunotsvaga unowanika pa pharmacy ipi mari yakati wobva wangoona mowonere atinongoita ma message ewhatsapp aya. Mungachida here chinhu chakadaro?

R (several): [Overtalk] Tinochida.

R: Unosvika uchingonodamburwa wasvika pauri ipapo.

R: Chavanofanirwa kuita ngavangoita percent iri normal kana ari ma bond kana ari ma US acho. Ngavachati chinhu chavakatenga dollar bond handiti US handiti kuma bond zvonzi 5 dollars.

I: Hoo rate yavo yakakwira?

R6: Exaclty. Chinhu ukachinzwa chichinzi 10 US haubvunze prize ye bond unobva wabuda ehe.

R9: Plus chiri kutinetsaka vari kutinetsa kuti US, US racho hatichatomboziva ne color yaro isu kuti tizive kuti ndere rudzii.

R: Ehe kana mushop haurichinji.

R9: Saka kana tava kunzi US tiri kuwomerwa hatina kwekutomboritsvaga. Munoziva kuti zvakaoma kuti une imwe mari muhomwe wombofamba nenyika yese uchida kumbotsvaga imwe mari, mari yekuti unofanira kunotenga imwe kuti uishandise izvozvo hazviite. Ngavaite zvinhi zvinogona ku affordwa nesu isusu ma Zimbabweans because tiri ma Zimbabweans yes, kana bond yacho iyoyo ichinzi ndoo mari yacho yemuno mu Zimbabwe ngatiishandisei iyoyo tigutsikane titenge mushonga nemari yacho yedu iyoyo yedu yatinoti yedu yacho because kana vava kutiti US, US munyika hatina saka tonoriwana kupi. Saka isusu tinenge tichiudza imimiwo vama pharmacy kuti imimi ndimi munogona kunotaura nevakuru vakuru kuti zvinhu zvakaoma izvi.

R6: Especially murwere we shuga. Murwere weshuga haijumbike hazviite nekuti hupenyu hwake hwatova pama piritsi.

R9: Ehe.

R: Ukaita two days vasina uri kudzoka kunooku mari yemubhedha hazvinei kuti anenge agara ma hours you need to pay kana two hundred and something you need to pay kuti murwere wako abude apa uri kuda kutsvaga mushonga wemapiritsi zvinhu zvinenge zvakaoma izvozvo pakurarama.

R9: Isusu ngatichengetedzai hupenyu hwevanhu saka tirikungodawo kuchengetedza hupenyu hwevanhu. Nekuti mukaona vezera ramai vangu ava vanoenda kupi kunotsvaga US ava vakagara apa ava? saka zvinenge zvakangooma.

I: Toda kuvhunza nyaya ye customer service pane mapharmacy anofunga kuti tikabata ma customer zvakanaka vanoramba vachidzoka ku pharmacy kwavo. Saka ndoda kubvunza kuti tomboti wawana ma pharmacy anenge ari kudhura nekwakacheaper asi kwakacheaper kwacho kuri busy havatauri newe kana vachitaura newe vanongoku roughura munonotenga kupi?

R9: Zvagara munhu zvagara munhu unongoda paunopihwa reception nekuti zvakafanana mazera ana mai vedu ava vanoda kusvika vachisvikonzwisisa nekuti mapiritsi avo chaivo mukavabvunza havagoni kuadeedza mazita avo.

I: Uhmm.

R9: Handiti saka iwewe urikusvikova attenda unofanora kusvikova nemoyo murefu nekuti uri kutanga kuvatsanagudzira kuti mbuya zvakamira zvakadai zvakadai saka ukavachenamira zvakadai mangwana vavodzokako here?

I: Saka kana wakacheaper munguva inoiyi zvinhu zvakaoma kudai?

R6: Kana kwakangocheaper unotongomira [overtalk]

R9: Unotopedzisira wakungozongoshingirira kuchenamirwa ikoko nekuti muhomwe ndoo murikukutuma [overtalk]

R6: Ende vari kuita chitsotsi emwedzi havaise emwedzi akakwana.

R (unidentifiable): Haaise ehe.

R6: I still remember ndichiuya muna February ndichiuya kuzotenga muna Mbuya Nehanda umu emwedzi vakandipa e half ndobva ndazouya panaapa avepo ndikatengeserwa e mwedzi wese. Ivo vakati anodimburwa ivo ndakasvika mumba ndikaverenga book ravo haadimburwi vasvika ne half month apa vandicharger mari yemwedzi wese.

I: Makadzokerako?

R6: Ndakadzokera vakandiudza kuti akatengesa haapo nhasi.Asi zvinenge zvakachengetwa muma computer. Zvikatovharana asi ndanga ndavapa mari yangu yavanga vandicharger.

I: Munoziva here kwamunogona kunoreva kana mabatwa zvisina kunaka mupharmacy?

R (several) : Hatizvizivi.

I: Ehe munogona kuti kana musina kufara nemabatirwa amaitwa mu pharmacy munogona kuenda ku Consumer Council vanoshanda nema clients nema pharmacy zvakare uye kana musina kufara nemushonga wamunenge matengeserwa either quality wawo hauna kumira mushe waexpire kana chii munogona futi kuno reporter panonzi pa MCAZ kuma Avenues pa corner pa Third na Baines pane blue and white building very big you can’t miss it ehe munono reva zvakadaro. Sometimes you won’t even get the pharmacist in trouble vanenge vachitoda kubatsirwa wo but zvakanaka kuti anenge apihwa mushonga usina kufanira ataure nekuti ndookuti vamwe vasazviitirwawo.

R: Uhmm.

I: Saka musafunga kuti mukaitirwa zviina kunaka mupharmacy zvavharana pane zvamunogona kuita kuti either kuti mari yenyu idzoke kana kuti muwonekwe kuti ma[overtalk] Saka mukatenga mushonga mu pharmacy zviya munomboitawo here pfungwa dzekuti pamwe waka expire, pamwe hauziriwo chaiwo chaiwo kana kuti munotongo trusta kuti kana mapinda mu pharmacy?

R9: Isusu tino trusta imimi ndimi makaenda kuchikoro chebasa iri inini handina kuenda kuchikoro.

I: Uhmm.

R9: Saka ndakasvikoti inini ndirikuda [overtalk]

R6: Especially pama piritsi [overtalk]

R9: Mapiritsi anenge ane expiry date [overtalk]: Anenge ari koo koo koo anenge achima counter achimaisa muka pepa onyora hake kuti acha expire 2025 inini chigaba handina kuchiona saka kuvimbika kwava kutoda kwamuri imimi

R10: Zvamuri kutaura imimi ka munhu atenga mapiritsi panofanira kuva ne board rinomira richitarisa kuti zvinhu zviri kutengeserwa vanhu ndizvo here.

I: Riripo board rinongoti…rinoita ma inspections but mu Zimbabwe muna ma pharmacy anodarika 600 board iri rine vanhu vasingapfuuri 100 saka havakwaisi kuzvika kwose saka vanoto relyer kuti kana mune zvama sangana nazvo kwamaenda mukavaudza kuti imi pharmacy yepa corner apo endai mumbonoona zviripo vanobva vaenda.

R10: Saka isusu tinoshanda the same pama pritis unogonakutenga mapiritsi sei ethree months akaiswa chi label chavo chiya chiya.

I: Uhmm.

R10: Saka iwewe paunoreacta ndoopaunofanira kuziva kuti mapiritsi aya..?

I: Ehe ukango reacter unofanira kudzokera kwawakamatenga wotaura kuti ndareacta ivo ndoovanoziva kuti vozodii vanogona kuti enda unoona chiremba.

R10: Ndookunga wakachengeta ma labels [overtalk]

R6: Munoziva zvinoita vemu pharmacy?

I: Vanoita sei vemu pharmacy?

R6: Ukaenda uchirwaraka havakushaire mushonga even iri wrong treatment pane pamwe pekuti mumwe munhu akaenda BP yake yakakwira vakati izvoni maybe urikuda kuita Meningitis obva apuhwa ma tablets e Meningitis iyo iri BP.

I: Mupharmacy?

R6: Ehe havatomboshaiwe mushonga wekukupa vanongokupa chero wavapa[overtalk]

R10: Inenge n’anga.

R6: Hautombobuda usina mushonga.

I: Taurai henyu mhamha.

R12: Inini question yangu iri pama tablets mamwe atinomwa anonzi ma Asprin anongori famous famous handiti.

I: Uhmm.

R12: Asi chinozondinetsa manje ndikapinda mune iyi pharmacy ndinonzwa vanoti take half.

I: Uhmm.

R12: Ukapinda mune imwe take quarter.

I: Uhmm.

R12: Ukapinda mune imwe one to two saka kuti ndichinyatso ziva the correct dosage manje.

I: Uhmm.

R12: Haa zvakutondinetsa kuti ndoita sei?

I: Ehe zvinenge zvichidepender kuti because Asprin inouya muma size akasiyana.

R12: Ehe.

I: Zvakafanana nekuti saga rehupfu rinogona kuita 5kg rinogona kuita 10kg.

R12: Ehe.

I: Saka imi munoda kutora 1kg mukaenda nechi packet che 5 munonzi torai quarter ye 1kg.

R12: Eee.

I: Mukaenda ne 1kg munonzi torai kadimbu saka zvinodependakuti iro guru racho rinorema zvakadini.

R12: Hoo vano consider huremu here?

I: Ehe.

R12: Kana kuti quantity yemushonga uri mupiritsi.

I: Ehe.

R12: Ma miligrams?

I: Ehe ndiwawo andiri kureva ndoohuremu hwandiri kutaura.

R12: Hoo ndoo hwamuri kutaura.

I: Ehe saka une Asprin tablet re 300 mg.

R12: Eee.

I: Iwe chiremba vakakunyorera kuti tora 75 unenge wakunzi tora quarter 75 ya pinda mu 300.

R12: Eee.

I: Asi dzimwe nguva ma 75 iwawa anongouya ari 75 rakadero inonzi Cardioasprin.

R12: Eee.

I: Vanonobva vakuti tora rese rakadaro riri 75, 1 ndopaunonzi tora 1 kana kuti tora quarter. zvaitokunetsai saka makambovhunza mubvunzo uyu here mu pharmacy zvamuri kuti nditore quarter ndakambonzi nditore one makambovhunza here?

R12: Eee ndakavavhunza.

I: Vakati kudii?

R12: Vanongoti endai munotora yatakupai iyoyii.

I: Havakutsanagurirei?

R12: Eee havanditsanangurire.

I: Havana nguva.

R12: Eee.

I: Vanogoita busy nei macustomer ari kuti hatina mari.

R13: Sister handiti koo pama prices standard rate yemu Zimbabwe is 1:250.

I: Uhmm.

R: Saka ndikatengeserwa ne rate ye 4 kana kuti ye 3 ndokwanisa ku reporter?

I: Hamukwanisika I business remunhu.

R14: Horaiti. I business remunhu koo kana maworkers acho akati kana ukatenga ne US racho hariiswe pa till rinoiswa pa side.

I: Makazviona zvichiitika here?

R(several): Zvinoitika kakawandisa.

I: Saka US hariiswe mu till?

R14: Hariiswe mu till.

R: Sa ka bond rinotoshanda because vanozonochinja US voisa bond umu.

R: Iye osara neyakewo muhomwe.

I: Kana muchiti hariiswi mu till munorevei pa reciet panenge pakanyorwei?

R: Panenge pakanyorwa mari ka apa.

R: Ma pharmacy emunoomu anongoda US asi US racho hamuna.

R: Reciept pamusoro rinenge rakanyora bond apa pasi apa US.

R: Ehe ndiwo ma prices acho.

R: Saka you can’t accuse them because iyewo anoti muchina wedu unongoprinta US ne bond iwe wabhadhara chii.

I: Asi hamunoni richiiswa mutill [overtalk]

R: Aiwa US rinoiswa pa side rinokosha ka iri.

R9: Mumapharmacy ndimo munotestwa vanhu shuga kana tiinavo mudzimba handiti iye zvinezvi hatichazvinzwisisi unoenda nawo wononzi 10 dollars kuswera mangwana wononzi 15 dollars kuswera mangwana wonzi 5 dollars.

R: Hatichatombozvinzwisisi nekuti vari kutesta nema US, BP chete ndiyo yave kungotorwa mahara shuga inobhadhariswa.

I: Chii chinobhadhariswa?

R: Iyoyo yekuti kana uchida ku testwa sugar, kana une ma bond 15 dollars [overtalk] Saka tochidii?

R: Haa kana pari ipapo ipoint zveshuwa[overtalk] munhu weshuga kana achida kufenda apa anokufendera hake.

R: Anokufendera havaite nyangwe akatofizuka kana pasina mari haveteste kuti vazive kuti vari papi [overtalk]

R: Kutozoita plan yekuvananzvisa shuga ivo vasingadye shuga yacho.

R: Iyezvinezvi zvakangofanana takabva tangotengawo zvimushini zvedu zvekugarawo nazvo kupi kumba, ndochienda nechimushini chiya chiya havatobvumi kushandisa chimushini chedu nekuti vanoda chavo kuti vandibhadahrise mari.

I: Munenge mune ma strips here?

R: Zvese zvinenge zviripo.

R: Muchina kana uchitengwa unetengwa une complete.

I: Saka munenge mune mastrips?

R: Ehe.

R: Chititesterai.

R: Chingotitesterai horaiti chindidzidzisai kuti kana ndiri kumba ndino testa sei aiwa munondiendesa ku jeri nekuti munenge muchindiitisa basa risiri renyu horaiti chindiitirai imimi havasi kuda.

I: Ahii! Mupharmacy vari kuramba kukudzidzisai kushandisa glucometer?

R: Exactly.

R: Ivo vanotengesa futi ma glucometer acho.

I: Ivo ma pharmacy acho.

R: Ehe.

R: Ivo ukavatenga kwavari ndookuti vakudzidzise but zvakafanana neni ini ndine ma changes ekuti ndinowana out of the country.

I: Uhmm.

R: Handitika Ndauya nayo chingondidzidzisa haabvumi.

R: But imimi mutori right mutori ready pamunouya nemuhu wenyu ari kurwara muno huyai nawo panopinda muchitorwa BP moti takatenga machine tidzidzisei kuushandisa.

I: Kuushandisa ehe. .

R: Because ku pharmacy vanogona kuda kukunetsai ka but kunooku ma doctor haamboramba kukudzidzisai.

R: Taatakutofunga kuti tava kutodzokera kuchikoro, totoenda ka kuchikoro kunodzidzira ikoko.

R: Nekuti kana une murwere weshuga ne BP unoda kugara uchiziva nguva dzese.

R: Kuti zvakamira sei.

R: Munoziva chii BP ndakatoona kuti yakasiyana neshuga inini ndiri munhu we BP handiti.

I: Ehe.

R: But Inini ndiga ndakagara ndinotozvinzwa kuti BP yangu iri kukwira ndotozvinzwa ndega but munhu we shuga unogona kunge urikutoseka naye usingasive kuti paurikuseka naye ipapo shuga iri kukwira zvekuti unozongoerekana usina munhu arikukupindura. Saka moziva kuti vanhu vakadaroka havasi vanhu wekuti kunyangwe dai ndasvika handina kana dollar munhu obva awomesa moyo wekuti handisi kutobata nekuti hapana mari saka chinokosha chii mari nehupenyu hwemuhu just kungotest? Handina kuti umupe tablet but kungotesta chete.

R: Ndakambonzwa vamwe nurse vachitaura apo vachiti vamwe venyu vari kuuya pano shuga dzakadzika vamwe vari kuuya shuga dzakakwirisa saka chindiudzai kuti kana zvichiitika tiri panaapa ko kuzoti kana tirikumba zvingani zvinoitika because tinouya ku review ka one pamwedzi, vamwe vanouya after three months zvakasiyana siyana zvinenge zvichiita sei. Saka unenge uchiti ukaenda kupharmacy vanokubatsira kuziva kuti zvinhu zvacho zvakamira sei izvozvi zvakamira sei.

R9: Because ndikaziva kuti yakakwira ndozviva kwekudzoka nako kuti regedza ndimhanye navo kupi ku hospital.

I: Munozivawo here nezve Association yema Diabetes ee Zimbabwe Diabetes Association makambonzwawo nezvayo?

R: Togoinzwa but hatii followi zvedu kuti tizive kuti inoita nezvei.

I: Association yevanhu vanosimbirwa nechirwere che Diabetes.

R: Kwairi?

I: Kana kuti inoita nezvei kana kuti makamboitrya kuiwona kuti inojoinwa sei.

R: Kudhara kuchina chinguwana taijoina ne dollar vachiri pa Annex vasati vaenda kwavari uko.

I: Hoo ok ndoopamaiziva nezvavo, saka ikozvino hamuchaenda?

R: Ndakasiyana nazvo ini.

**Text F**

R1: Inini dambudziko ndirori re shuga. […] Asi pari zvino chiri kundinetsa, ibvi. Ndiyo imwe hosha yandiinayo [….] kune mapritsi here anoita kuti panondirwadza kudaiwo, papere kuzoti panyarare, parege kuita kurwadza kana kuti kuzvimba.

I: Mishonga inorapa kurwadziwa izere iriko, mukatsanangurira chiremba zvamanditsanangurira izvozvi, vanokunyorerai pasi zvinoita kuti ibvi renyu risakurwadzai.

R: zvekuti ndingazouwana here pano?

I: papharmacy iyi?

R: ehe

I:Ehe zvinozodepender kuti manyorerwa kuti kudii ndoozvandanga ndichida kuhwisisa kutimuri kuiwana here kana muchinge mainyrerwa papharacy yedu iyi?

R:Kuri kuti taishaya tozoita sei?

I: kana maishaya munosiita sei?

R: ehe kana taishaya toita sei? Motiwo kana maishaya moenda kwakati so so mungaiwana.

I: Hoo munenge muchida kuudzwa kwekuti munogona kuiwana?

R:Ehee.

I: Ehooo

R: zvekuti hazviite here kuti – isu sister ndezvekuti ka isu vamwe togara kure, hatisi togara muno mutown, togare kure, tobvisa dzimari inaudible]

I: munenge maakuenda zve kunotenga mu pharmacy, asi mupharmacy imomo kuri kuti maushaya munokwanisa kubvunza pharmacist vari imomo kuti hakuna kumwe here kwandingaenda, nekuti vane ma group avo avanoungana mumaWhatsapp umu vanoudzana kuti ndiani ane chakati ndiani ane chakati. Vanogona kukubvunzirai 3 minutes vokuudzai kuti mukaenda pakati, munonomuwana. Asi munofanira kuvabvunza nekuti kana vari busy vanogona kungoti hatina, votokuregai muchiinda, asi mukabvunza kuti mwanagu hauna here kumwe kwaunoziva kushamwari dzako, vanawo, anokutsvagirai. 3 minutes chaidzo

R1: ndoozvatanga tichida kuti tinzwe

I: Hooo. Taurai henyu mhai

R2: Iiii mishonga tiri kumwa shuwa asi iri kudhura, hamunawo here kwamunoziva kwakachipawo? Taane makore tichimwa mishonga yeshuga but mari dzacho hatichadzigona dzaakudhura, zvakanyanyisa

I: dziri kudhura dziri mumabond kana dziri mumaUS?

R2: Dziri mumaUS imomo but US yacho hatisi kuibata. Bond racho iroro kuti riwanikwe, mumwe waunoda unbova wanzi 45, uyu wakazoti, apa unomwa mhando 4 dzaunomwa. Kuti uibuditse manje ipapo, zvakaoma. Hapanawo here kwamunoziva kwakachipa?

I: zvandanga ndichitsanangurira baba kuti munogona kuudzwa kwakawanda kwairi, munogona kuudzwa futi ma prices mobva macompaya musati mafamba. Kunenge kwaakutobvunza kuti ko kana ukandibvunzirawo, kana 3 mapharmacy, unotoona musiyano pamwe wakatokura. Mobva mangoudzwa kwekuiwana. Asi zvinenge zvakangonakawo kuti iwanikwe …

R2: Iwanikwe panapa handitika? Haiwa tinogona kusvika ipapo.

R3: Ndoda kubvunzawo kuti vatiri kunzwa kuti vari kuita zvema herbal, muri kuvaonawo sei? vari kutaura zvema herbal, kuti mukange muchishandisa ma herb masugar levels anokwanisa kudzikira?

I: mushonga iri… inonetsa pakuti inofanira kutanga yatestwa kwemakore, handiti, isati yaziikanwa kunzi inoita basa rei chaizvo chaizvo. Mukaona painotengeswa paya, even label rinenge rakanyorwa, hazvibvumidzwi kuti inyore claim, kana isina kutestwa. Ndosaka muchiona kuti even pakutengesa kwavanoita vanhu vema herbal, havaiti zvekushambadza pachena, hamumbofi makaiwana yakazara maOK, kana kuti yakazara mu pharmacy yakanyorwa zvamuri kutaura izvi, nekuti..

R3: But some people are advertising ma herbs iyayo, even pamaMedia.

I: Ehe vanotodaro because havabatwe, zvakangofanana nekuti munogona kutowana munhu achitengesa mishonga yake muroad, akabata megaphone , vanozviita asi hazvifaniri kudero, kungoti pane organisation inonzi MCAZ, inofanira kunge ichisunga vanhu ivava, vari short-staffed, saka kuti vaende kwese ikoko vachinovharisa zvese zviri kuitika izvi, vanenge vaakutoprioritiser kwekuenda otherwise hazvifaniri kunge zvichidero. Zvinofanira kunge zvaongororwa kwemakore, ndosaka muchiona paya pakambotaurwa kuti chakati chinorapa cancer chinodii, government yakakurumidza kuti yowe yowe nyararai, nekuti zvinofanira kutanga zvatestwa.

R3: But pane hurongwa here hwamuri kutiitira kuti munge muchitiitirawo safeguard kuti tisazongotanga kupihwa mishonga iyi, nekuti mishonga iyi zveshuwa inenge yakachipa

I: mmmm. Pane register yemishonga yema herbs ari approved – kungoti zvinowanikwa pa internet zvinhu izvi, zvinenge zvakanaka kuti zviwanikwe kwese kwese, but vane website vandiri kutaura ava, vanoita basa irori vane website inotori ne list yemishonga, ma herbal iwawo, akabvumidzwa kutengeswa. Saka mukaona mushonga wamuri kuda kutengeserwa usipo pa register iri, munofanira kumboitawo kaungwaru mbichana.

R3: Ah maita basa.

I: Pane vamwe vari kuda kutaura? Ndaona kuti ava vava paphone but he wanted to say something… Hello.

R4: How are you? Ndoda kubvunza kuti ma sugar testing strips aya hakuna here kuti anongopukutwa zvawo, because ari kudhura. Hakuna here ekuti you can reuse again wamboshandisa?

I: Handisati ndaaona. Because problem ndeyekuti, pamunoisa ropa renyu paya paya, rinoreacter nesomething chiri imomo kuti chiproducer reading. Saka once character, chatopera.

R4: Horaiti

I: Eeee. Zvakafanana ne surf kana muchiwacha paya, hanti mukwacha mvura inobva yatopera simba? Ndoozvazvangofananawo neizvozvo. Asi it’s a good idea actually. It would be very innovative for someone to find out… kuinventer something that’s reusable.

R4: Ari kudhura.

R5: Mastrips they are too expensive.

I: And they are very specific to your machine ka, if you have a particular machine…

R5: Right. Saka because of that rega ndione pamwe I will invent something.

I: It would be interesting to talk to a biochemist and find out actually kuti hapana here anongona- it’s a very good idea actually. But I have not seen them yet. But if I do, I will endeavour to put a notice. Next time you come you will see the notice. Pane vamwe here vane zvekutaura? Ndamboona ruoko rwenyu rwanga ruri up…

R6: Handizive here kuti pane ambobvunzawo mubvunzo uyu, ndazenge ndaapa phone. Ko like baba vangu vari kuzvibaya, ma injection. Pane possibility here yekuti vambozoregera mainjection vaende pamatablets?

I: usually kana watoenda pa Insulin, kudzokera – unotobva pamapiritsi uchienda ku insulin. It’s a higher step up. It’s very unlikely kuti vanozokwanisa kudzokera – but zvinenge zvichida chiremba vachiongorora, vachiona ma records avo but hapana wandati ndamboona akati ambobva pamapritsi akaenda pa insulin akazodzokera kumapiritsi.

R6: Hoo?

I: Kana matova pa insulin,

R6: Vakatanga ne insulin,

I: Saka hoo yaa I am not optimistic but munogona henyu kubvunza chiremba I suppose.

R6: Last time vakamboti hazviite kuchinja.

I: ehe. Munhu watoenda pa insulin watova pa insulin. Saka vanozvibaya nemasyringe? Vanoita sei? Mapenset?

R6: Masyringe

I: Zvaakuvarwadza?

R6: Ehe I’m susre zvinozosvika pakurwadza but panenge pasina zvekuita. Pane imwe way here besides syringe?

I: pane ma penset. Anoti dhurei zvawo. Inoita kunge biro. Kuita kunge biro yekunyoresa, chaiyo. Kungozvidai. Ine tsono but haiite se needle, haizoite zvekuzvibaya mabairo avanoita. Asi inoti dhurei. Asi it’s easier to use, ndoona vanoishandisa vanoti- vakaishaya iyoyo vanotorwadziwa nekutenga tsono havadi.

R6: inowanzoita marii iyoyo?

I: Zvinodepender kuti chiremba vanyora kuti kudii. Dzinouya dzakasiyana siyana dzine madifferent types of insulins saka zvinodepender kuti chiremba vanyora kuti chii.

R6: hoo type ye insulin yacho?

I: ehe, dzimwe ndedzekuti munhu akazvibaya, anoswera zuva rese, dzimwe ndedzekuzvibaya munhu achangodya, dzimwe ndedzekuzvibaya ka three pazuva. Saka zvodepender kuti anyorerwa yakadii.

R6: syringe ndeye once a day.

I: zvodepender kuti anyorerwa sei but ma pensets anoti dhurei kupfuura ma syringe asi ari nyore zvawo kushandisa, vana chaivo vanogona kuashandisa. And it’s less painful.

R6: hoo it’s less painful?

I: Ehe and it’s easier kuzvidoser. Hanti kana vachizvibaya ne syringe vanoita zvekudhonza voverenga vodii, kana light risina kumira mushe vombo- but penset haina izvozvo, kungozvibaya.

R6: Hoo but ingori…unongouser ka1? Penset? Ingori one dose chete?

I: Unenge watomeasurer, kungotenderedza tenderedza kusvika wasvika pa number yauri kuda wotozvibaya. Ehe it’s quite easy.

R6: Hoo ho right.

I: Ok. Pane vamwe here zvavanoda kutaura zvakavagara pamoyo pavo kuti haaa izvi zvikasagadziriswa izvi haa…? Yes.

R7: Ndimbotaurawo. PaPharmacy yepanaapa haikwanise kuramba iri equipped here nemishonga yacho? Yepano iyoyi?

I: zvoda mari. I think ndoozvanetsa izvozvo kuti hurumende inogara ichingoti haina mari saka kuti irambe ichingostocker pharmacy- haisi yega yepano. Mapharmacy eku Harare hospital, ekupi,

R7: Enyika yese

I: vanongotambura nekushaya mishonga ehe. Asi ndoozvazveezvichida kuti vanhu kana vauya pano vanyoregwa mishonga yavo vongoenda pacorner apo vopiwa voenda.

R7: saka mishonga inogadzirwa kunze kwenyika yese?

I: Mizhinji yacho yese ehe. Inotogadzirwa kunze

R7: hatina yatinogadzirawo muno? Especially yeshuga iyi?

I: haa, mishoma inogadzirwa muno. Pamishonga yese inoshandiswa muno ndofunga 20% ndooinogadzirwa muno but vanogadzira muno vacho, zvavanogadzirisa, vanozvitenga kunze. Saka you find kuti zvese zvinongoda kutengwa,

R7: hoo. haa dai vatora mari yema diamonds vongoisa muzvipatara zvaibatsira.

I: muchiti ichiko?

R7: Ndaiti dai vatora mari yemadiamonds ese vachitenga mishonga yemuchipatara zvaibatsira.

I: Ichiriko here mari yema diamonds?

R7: Pane kutenga mazimotikari vachipedza havo mari vachitadza kutenga mishonga. Iriko but vanoiisaka muhomwe dzavo. minister of health ari kufamba nezimota rinodhura zvisingaite but kuzvipatara kwacho hakuna mishonga. Vanhu vari kutambura mishonga vari kufa nekushaya mishonga. Haa it’s a very bad idea, kana muchivakwanisa kuvaudza kuti imboitai muchiona, matengerewo amunoita mota aya. Ukatarisa mot adze ministry of health ka, dziri very expensive dzakawanda. Tisingatauri ministry of health chete but in particular tiri kutaura ministry of health, dzakawanda. Imi ma nurse acho hamuna zvekunyatsopfeka zvekushandisa, mishonga yacho hamuna but kuri kutengwa mota dziri expensive dziri kufamba muroad umu. Hazvibatsiri chinhu.

I: Vanogona ka kutaura kuti mota dziri kubva kuma donor.

R7: Madonor acho why asingaprioritiser mishonga yacho inozoshandiswa nemunhu ari kushandisa mota?

I: madonor anonzi anoprioritiser mishonga ye HIV ne TB ne Malaria. Zvinopomeranwa zviya. Manje diabetes haipomeranwe.

R7: But manje handiti inonetsa kurapa?

I: Ehe inonetsa.

R7: Haa horaiti maita basa.

I: Maita henyu nezvamataura

**Text G**

I: Asi munosingoitenga somewhere else? KwaMutare, mabva kwaMutare?

R1: Ehe

I: Hiii ndimi vemvura zve? Ndimi vekunaiwa [chatter about cyclone Idai unrelated to the research].

R2: Tauya namuchembere ava haa mishonga yavo haiwanike pano.

I: Heya hamusati mamboiwana pano? Saka kwamuoiwana inowanikwa zviri nyore?

R2: Haa hazvisi nyore. Process yacho yakanyanya.

I: hindava? Inodhura here kana kuti iri long? What is particularly the problem?

R2: Yaa it’s long hanzi womboenda, unofanira- dzokera unonyorerwa section 75, wosvika chiremba haapo, abuda, aita sei..

I: munoinzwisisa here mashandiro ayo section 75 kana kuti basa rayo nderei?

R2: Takatanga kuzongoda kufungidzira from there vachiti inoordwa kunze what what

I: Ehe, kana mushonga yamunoneeder usina kuregistwa kana kunyoreswa muno inofanira kutengwa kunze saka vanoda kuona form iroro kuti vazive kuti zvedi maishaiwa here muno munyika, mashaya alternative here vokupa mvumo yapharmacist kuti anokutengerai kunze.

R2: Horaiti saka haaa Then after that kana yazodaro then yozodhura, haa inenge ichidhura zvayo.

I: Ehe kana yatengwa kunze inenge yaakuto chargwawo nema US

R2: Ehe ndizvozvo saka generally ndizvozvo hazvo.

I: Ahh it’s alright. Ko mhai mune zvamunodawo kutaura?

R3: Aiwa tiri tese hedu

I: Ohh mauya mose? Ahh inga mavaperekedza zvechokwadi. That’s nice. I hope vanonaya.

**Text H**

R1: Pamishonga yeshuga, moona zvandiri inini ndinonwa 2 ma types. Glibenclamide ne Metformin. Mawaniro atinoita, mumwe musi unogona kuti, kunodiwa maUSA. Handiti? Kana usina USA unenge uchinzi maybe pa 4 dollars yebond uri kutenga mapiritsi 15. Watenga mapiritsi 15, ndeendisisina panguva iyyoyo pekuti mapiritsi andiperera panguva yekuti handina mari yakakwana yekuti nditenge anopera emwedzi saka, kutenga kwatinoita kuya, mumwe musi unnzi 4 dollars, mumwe musi wonzi 3 dollars, mumwe musi unosvika mamwe mapharmacy obva ati USA chairo chairo. Saka zvimwe zvacho zviri kutiomera ndezvekuti mari yekuti nditenge kamwe chete, nekuti ini ndomwa mapiritsi akati akati uwandu, ndotenga ka1, mari iyoyo iri kunetsa kuita sei, kubatika. Saka tazotenga ma half half, ndoopaunozodefaulter mamwe acho sometimes nekuti nhasi apera, kunoti pharmacy ino, hanzi aperawo metformin hapana pane glibenclamide chete. Ndaakuchitenga iripo. Ndomwa iripo but ndakanganwa kuziva kuti iyi icourse inofanira kumwiwa yakaita sei, yakakwanirana. Saka zvimwe zvacho tiri kukonzereswa nekushaya. Eheee

[chatter from new entrant into focus group]

I: taurai mhai

R2: maStrips ndooari kutidhurira, mastrips ari kudhura,zvekuti uyu ndofanirwa kumubaya makuseni nemanheru, ndisati ndamubaya ndofanirwa kumutesta kuti ndione kuti iri sei. Saka you find kuti nekuoma kwaita zvinhu, ndanga ndakungomubaya makuseni ndichitya kuti ndikamubaya manheru, pamwe yakadzika, otindiitira zvimwe.

I: Okk

R2: Taichimboatenga kudhara kana kuma 16 dollars but ikozvino izvi mari dzacho, mapharmacy acho, maBond nemaUSA, eish. Zvakaoma

I: taurai henyu mhai

R3: Zvimwechetezvo. Inini ndinotobaya insulin. Saka zvakatooma kana uchinge usina, US. Kuti utenge kana ukaenda kupharmacy unonzi huya neUS kuti utenge. Pamwechete nemaStrips ari kutaurwa nevava iii anonetsa kutoti uteste zvinofanirwa zvacho kuti before or after 2 hours chii chii unotoona kutoti ukadaro unozoita imwe nguva refu usina. Fanika inini mari yacho iri kunetsa zvekuti vari kuSouth vakasanditumira ndeendichishupika. Saka dambudziko riri pamari, mishonga iri kudhura.

I: iri kudhura irimo mushop here kana kuti, vanogona kuti hatina chero une mari yako?

R1 and 3: dzimwe nguva vanoti hakuna. Ehe dzimwe nguva kunenge kusina. Dzimwe nguva unoiwana, asi nguva zhinji kana une US unowana.

I: hooo. Taurai henyu baba

R4: Iyo nyaya yekuti kuuye maUS kuuye bond iyi caused confusion munyika muno. Quite honestly, isusu we are very sore we are highly disadvantaged. [chatter about cups etc. somewhere in the vicinity]

R4: Yaa so ndingati now I don’t know where to address it. The government kana kuti matongerwo enyika akatikuwadza nenyaya yekuisa bond US iyoyi. Yaa asi iyezvinozvi – asi pavakaunza bond vaingoti I 1:1; but look, zvakadaro so zvaingoratidza kuti handizvo ba nekuti vanhu vaitoti tiri kutora ne ma USA, so if I don’t get maUS, then mushonga ndinoushaya. And mishonga iri kunetsa like inini ndine dosage ye, ye inonzi chii? Galvus 50/1000, Unoziva kuti haipo, handina kumboona shop yairi. And then the substitution yandakaitirwa na pharmacist, ndeyekuti take, 50 by 500 yeGalvus ka, then ndozotora imwe iri 500mg. That’s how I have done it for this month because 50/1000 is not available wherever you go. Ndakatenderera. Nditori nemotikari, munondiona ndichifamba netsoka, mota ndinayo. asi ndakasvika kunaana Newlands kupi kupi, 50/1000 is not available.

I: Makamboti here pharmacist vakubvunzirei pamawhatsapp group avari mamember nekuti vanenge vari mumwhatsapp group ka, vanogona kungoisa message kuti pane Galvus 50/1000 here. Anenge achitaura nevanhu vakati wandei ka mugroup. Then kana anenge anayo anogona kungoti ndinayo. Saka dzimwe nnguva munogona kuvapa suggestion iyoyo.

R4: Haa vanotongokuti try Trinity pharmacy, try Kensington, try, haa ndoozvavanotoita,

[chatter]

R5:Zvazotaurwawo na Mama. Ndine hama ari kuzvibaya. Inini ndaandisati ndapinda hangu pakuzvibaya but I think I will get there soon. Mama ndine anozvibaya it’s the same problem. Haiwanike or iri kuwanika ichidemander US explicitly such that munhu unotofaira kuenda kuroadport, unofanira kupinda mustreet woti ndiri kutsvagawo USA. And kazhinji kacho you can be very unfortunate ka imhosva ka? That’s illegal dealing, money laundering, unosungwa. Hazvina justification before the court of law ka.

I: Eh kana watongoita mhosva waita mhosva.

R5: Hazvina justification

I: ko ma medical aid ari kufamba sei?

R4: Now, medical aid, sorry let me talk a bit, medical aid zvayo, enda kumapharmacy ikoko, anotaura kuti we are not taking medical aid. Now iwewe tenga, then you claim from the medical aid. Now medical aid ndakazviona like my wife paanga pane mushonga waakatenga like that. Now medical aid tiri kuti unenge watenga – handiti pane US pane bond?

I: Yes

R4: Now, medical aid fanika CIMAS, iri pa 1:1 haina kuratesa, haina ku rater. Saka ukatenga ma tablets e 40 dollars, now US equivalent can be what, can be 80 dollars. Then CIMAS can refund you 40 dollars, yopinda mu bank yatova bond. Haisi US. That’s what is happening ne medical aid. Useless.

R2: Isu hedu, Ini hangu handina medical aid. Ndinongoshanda ne cash.

R4: Ndoozviri kuitika izvozvo. Haa mapharmacy akawanda ari kuti hatitore medical aid.

R3: Ndanga ndichida kutotaura kuti chimwe chinhu chiri kuitika, ukaenda ne medical aid uchida mishonga, kwacho kwaunonzi enda madrugs anenge ari out of stock.

R4: Eheee

R3: Ukabata USA, the drugs are in stock. Saka medical aid yes, we have got ma medical aids, but hapana kwaunowana mushonga wacho nemedical aid kunenge kusina. Seni ndinomwa mapiritsi eBP. HCT, inoita dollar, ndiyo yandinowana pa medical aid. Aya ekuma 30 dollars aya, anenge ari out of stock.

R4: Mazviona manje.

R5: Chimwe chandakaonawo pama medical aid ka, kubva pakatanga confusion yema RTG what what, vanonyeba kuti tiri kutora handiti, iwewe wotofillisiwa form, but price yaunotenga nayo ka, pamedical aid, inongofanana, neimwe pharmacy unongoudzwa same price. Drug riri kuita 19 dollars, ikoko unenge waakunzi shortfall I 19 dollars. Saka handizivi kuti hatisi kunyudzwa here kumapharmacy aya kana kuti chii chiri kuitika.

R4:Muri kunyudzwa.

R5: Senge yedu Premier iyi, unongonzi ehe tinotora, wotofilla asi price ukabvunza kune imwewo pharmacy, price ndoimweyo. Saka you wonder now kuti what’s happening.

R6: Pamwe Premier inotori nani, Ini ndakanga ndine Corporate 24 ndakatodzikana ndikarega. Nekuti waiti uku uchibhadhara medical aid, ukaenda chero nyangwe kuParirenyatwa chaiko chaiko, vairamba voti iwewe bhadhara wozoclaimer. Pakuclaimer kwacho ipapo pane dambudziko, hapana chaunombowana inongofa kwako. Kusvika ndatoirega medical aid iyoyo. Kuti haa zviri nani ndichingomira zvangu ne cash. Zvinhu zvacho zvinongoomera isusu varwere.

R4: Isusu vanhu vane chronic illness we are at a very big disadvantage. And we are going into the situation yakafanana neyevanhu vafa mu flood avo [reference to cyclone Idai]. I am telling you, that can happen. That disaster can happen to us because mari dzacho munyika hamuna, hapana mari.

I: Makambozviorganiser here semapatients kuti muite yatinoti lobbying kana kunotaura nehurumende muri boka?

R4: Havanzwi vanhu vacho

R6: Yes, vanoenda. Zvinoitikaka ndezvekuti – sorry zvimwe ndoita ndichidairira-

I: It’s ok.

R6: I see myself as a diabetic patient because pamwe pacho ka pane vanotaurira- voice of the voiceless. As Zimbabwe Diabetes association, kutanga kuchati, kumaMinistry kwacho kuchine vamwe, several times, kuchiendwa kunoapporacher. Aim yacho yanga iri yekuti, we have got machronic conditions ari kurapwa mahara muno. But why can’t you say kuti diabetes ipindewo ipapo because diabetes iri kutodhura kuirapisa than TB. Vanhu veHIV, do you know kuti mabatirwo anoitwa HIV, naana diabetes, hapana anoapplyer. Mwana anongona kungozvarwa ari diabetic. Mwana anogona kungozvarwa ari positive. Handiti? Unogona usina kana musikanzwa yawaita kuti uve positive. But you end up waa positive. Zvimwechete ne diabetes, handiti. Like this young girl, akaapplyer here? Haana. Ko sei isingaitwewo diabetes- takaenda taendazve zvichingonzi, it’s in the pipeline kuti vazoti diabetes itreatwe for free. But hapana chati chanyatso… Saka ministry kubvira maminister achiri akabva kusvika varipo iyezvino, vari kuzviziva kuti chichemo ichocho chiripo. Pese kana maimboteerera paienda Dr. Mangwiro, vachingori kuZDA vasati vava deputy minister, chero vari pa TV, chero tikaenda ku awareness, taigara tichitaura kuti diabetes ngaiitwewo sezviri kuitwa mamwe ma conditions akaita seTB ne HIV. I remember ukaenda kuWilkins, I think unobhadhara card here kana kuti haubhadhare, kungodhindirwa card, wopihwa supply yako yemwedzi. But look at the diabetics. Munhu we HIV ndikanzi ndiri HIV positive nhasi, kuda kwangu kuzocheka futi kuti ndiri positive here because ndakatoudzwa. And viral load maybe it’s once a year, but ma diabetics we do maHbA1c every 3 months. Tinoita U and E tinoita urinalysis then they are supposed to check ma blood sugar level about 6-7 times a day. Ndoine mastrips ari kudhura. Hainei nekuti sugar yanga yakadzika kuseni. Sugar yanga yazokwira kuseni, no. Tinenge tiri kuti by right, vari supposed kuti just before breakfast, two hours after. Touya pama drugs avo, madrugs ediabetes ari kudhura. Nekudhura ikoko haasi kuwanika. We have got insulin like Lantus. Tanzwa nekubvunzwa kuti Lantus toiwana kupi? Haisi kuwanika Lantus. tine Glargine, tine Tresiba, iri kusubstituter iwayo. It’s Very expensive. Last time pane akandiudza akati, Sister, drug iri ndarinzi 102 bond.

R3: Kana kudarika

R6: But that 102, is for one drug and usually iri kuenderana neMetformin nemaStrips. You see kuti pazviri kudhurira. Zvichemo izvozvo kuMinistry zvakasvika. Hatigone kuenda hedu tese kuti ngatirongei mudungwe tiende kuministry but vacho vanotimirira vachitaura, zvichemo zvinosvika.

R4: Hoo

R6: Even pamwe pacho mukateerera, pane pandinotoziva kutinhasi kwataurwa zvakati regai ndimboteerera. Zvinobuda pa news. Mumapepa zvichibuda.

R4: But why are we falling short sister ****? Our patron is the deputy minister surely…

R6: Vari kushingaira. That’s why muri kuona kuti he built his own pharmacy, handiti? Kuti ivo, donzvo rekuti chiremba vazovaka – this pharmacy is mainly for ma diabetics iwayo because vari kuti vakawanawo mishonga iri cheaper, vanhu vanenge vauyawo vachirapwa pano, vobva vawana mishonga iri cheaper. Vanoda havo kuenda kumamwe maprivate izvowo hatinga… but itori advantage, yekuti – nddovainyanya kutaura nezvazvo, vachinonetsana naanaGwinji naanaParirenyatwa. Now vaamu system saka isusu tiri kuti zvaakutonakirawo vakawanda because ma chronic conditions aya – yesterday chaiyo, we were busy kutsvaga mamwe madrugs acho ari kuda kunzi astocke up. Saka dambudziko rimwe riripo, munyika medu muno, some of the drugs hamuna.

R4: Hamuna ehe

R6: Go to Norvo Nordisk. Handiti munoziva kuti ndiyo inosupplier Novo insulins. Talk to Mr Mutiro, he was saying, Zimbabwe yasara ne stock ye 2 weeks. Which is last week ne this week. Which means pakasawanika mari nekukasika, yekuorder those drugs, tichatoita futi, shortage yakanyanyisa, yema insulin. Ana Novorapid, Protaphane, Levimir, Actrapid. Very soon pakasaallocatwa mari imwe, very soon. Vaitozvitaura kuti nyika haisisina ma Insulin. Mudiabetic, anorelyer neinsulin. Ihove iri mumvura. Hatigone kuiburitsa mumvura saka those are the challenges.

R4: And they are big challenges, but we can – like I SAID, I felt kuti our patron being a diabetologist and a deputy minister, we’ll have…

R6: He is trying his best, very soon

R4: Ndakatofara when he was appointed deputy minister ndichiti now our problem is resolved.

R6: Yes it’s being solved.

R4: Ndichiti nhamo yapera.

R6: Iri kupera very soon.

R4: But we seem to be getting worse. Ndaverenga mu paper headline, hanzi petrol yasara ye 4 weeks chete? And hapana mari yekuorder imwe. [laughs]. Petrol iri available it will last the country 4 weeks at current consumption. Handizive [indistinct chatter]

I: Unless kana pane vamwe vane zvavari kuda kuwedzera havo, I think ndatonzwa zvandanga ndichida kunzwa. Maita henyu.

[off the record discussion ensues]
